# A novel loss-of-function mutation of *PBK* associated with human kidney stone disease

**DOI:** 10.1038/s41598-020-66936-4

**Published:** 2020-06-24

**Authors:** Choochai Nettuwakul, Nunghathai Sawasdee, Oranud Praditsap, Nanyawan Rungroj, Arnat Pasena, Thanyaporn Dechtawewat, Nipaporn Deejai, Suchai Sritippayawan, Santi Rojsatapong, Wipada Chaowagul, Pa-thai Yenchitsomanus

**Affiliations:** 1 Division of Molecular Medicine, Research Department, Faculty of Medicine Siriraj Hospital, Mahidol University, Bangkok, Thailand; 2 Division of Medical Genetics Research and Laboratory, Research Department, Faculty of Medicine Siriraj Hospital, Mahidol University, Bangkok, Thailand; 3 Division of Nephrology, Department of Medicine, Faculty of Medicine Siriraj Hospital, Mahidol University, Bangkok, Thailand; 4 Sappasithiprasong Hospital, Ubon Ratchathani, Thailand

**Keywords:** Genetic techniques, Genomic analysis, Immunological techniques, Biological techniques, DNA sequencing, Next-generation sequencing, Genetics, Gene expression, Genomics, Medical genetics, Mutation

## Abstract

Kidney stone disease (KSD) is a prevalent disorder that causes human morbidity worldwide. The etiology of KSD is heterogeneous, ranging from monogenic defect to complex interaction between genetic and environmental factors. Since mutations of genes responsible for KSD in a majority of families are still unknown, our group is identifying mutations of these genes by means of genomic and genetic analyses. In this study, we identified a novel loss-of-function mutation of *PBK*, encoding the PDZ binding kinase, that was found to be associated with KSD in an affected Thai family. Glycine (Gly) substituted by arginine (Arg) at position 43 (p.Gly43Arg) in *PBK* cosegregated with the disease in affected members of this family, but was absent in 180 normal control subjects from the same local population. Gly43 is highly evolutionarily conserved in vertebrates, and its substitution affects protein structure by alterations in H-bond forming patterns. This p.Gly43Arg substitution results in instability of the variant PBK protein as examined in HEK293T cells. The variant PBK protein (p.Gly43Arg) demonstrated decreased kinase activity to phosphorylate p38 MAPK as analyzed by immunoblotting and antibody microarray techniques. Taken together, these findings suggest a possible new mechanism of KSD associated with pathogenic *PBK* variation.

## Introduction

Human kidney stone disease (KSD) is a group of common disorders that cause morbidity in human populations and a global public health burden^1^. The causes of KSD are heterogeneous, ranging from single-gene defect to complex combination of genetic and environmental factors^2^. The majority of kidney stones (~80%) are composed of calcium oxalate (CaOX) and calcium phosphate (CaP)^3,4^. Intrarenal and urinary ion imbalance is the known risk factor for KSD^5^. Growth of calcium oxalate stone over Randall’s plaque site appears to be the predominance of stone formation for the patients with hypercalciuria^6,7^. Exposure of renal tubular cells to oxalate, calcium oxalate monohydrate (COM), and CaP causes increases in reactive oxygen species (ROS) production and oxidative stress leading to cell injury and inflammation. These accelerate crystal formation, growth, and aggregation, which ultimately leads to stone formation^8,9^.

Previous studies using family-based or case-control analyses identified genetic variations of several genes in KSD, including variations in *calcitonin receptor (CTR)*^10^, *vitamin D receptor (VDR)*^11,12^, *calcium-sensing receptor (CaSR)*^13^ and *osteopontin (OPN)*^14,15^. Genetic variations in *claudin 14* (*CLDN14*), which encodes for the tight junction protein, was also discovered by a high-throughput genome-wide association study (GWAS)^16^. Our group previously reported KSD in Northeastern Thailand where the prevalence of this disease is high. The relative risk of KSD among the members of the affected families was greater than that of the general population (λ_R_ = 3.18), which suggests the genetic contribution to kidney stone in the Northeastern Thai population^17^. We also found genetic variations to be associated with KSD risk in Thai patients in case-control studies^18–21^. However, these common variants were likely a modifying factor, not a disease-causing factor. Genome-wide linkage analysis and exome sequencing were later employed to identify a disease-causing gene in families affected by KSD. These analyses showed loss-of-function alterations of *SCN10A*, encoding Na_V_1.8 α subunit of voltage-gated sodium channel, to be associated with KSD in the families studied. Na_V_1.8 was initially reported to be preferentially expressed in peripheral sensory neurons and heart tissue. Gain-of-function alterations of *SCN10A* cause painful peripheral neuropathy^22^, while its loss-of-function alterations contribute to prolonged cardiac conduction disease and Brugada syndrome^23,24^. In our study, we found that Na_V_1.8 α subunit protein expressed in proximal tubules and collecting ducts of nephron in human kidney. The variant protein (p.N909K and p.K1809R in the same allele) expressed in cultured cells was unstable, resulting in reduced current density as analyzed by whole-cell patch clamp technique. We propose that Na_V_1.8 channel may be activated by the depolarization of Na^+^ and help to increase Na^+^ reabsorption^25^.

In the present work, we continued our search for disease-causing genes in KSD via exome sequencing and genetic analysis in a different family that is affected by KSD. We discovered that a novel loss-of-function alteration (p.Gly43Arg, or p.G43R) in *PBK*, encoding the PDZ binding kinase, cosegregated with KSD in affected members of this family. The p.Gly43Arg substitution results in instability of PBK, which is a member of the mitogen-activated protein kinase kinase (MAPKK) family^26–28^, and the activated form of PBK phosphorylates p38 MAPK^26,29^. These findings suggest a not previously reported potentially new mechanism of KSD associated with *PBK* alteration.

## Results

### Subjects and clinical study

We recruited patients and members of 180 families affected with KSD for genetic study. The disease in these patients and the members of their families was not secondary to all known causes, including renal tubular acidosis, primary hyperparathyroidism, inflammatory bowel disease, Cushing disease, hyperthyroidism, or drug-induced KSD, as diagnosed by clinical history and symptoms, physical and laboratory examinations, acute acid loading test, and serum electrolytes. The normal control subjects included in this study (n = 180) were recruited from the same geographical area (Ubon Ratchathani Province in Northeastern Thailand) that the KSD patients and their families were recruited from. Normal controls and study group subjects were similarly examined, and controls were investigated by radiography of kidney-ureter-bladder (KUB) to confirm the absence of kidney stone. Gender and age data of study subjects and normal controls are shown in Supplementary Table S1.

**Figure 1:**
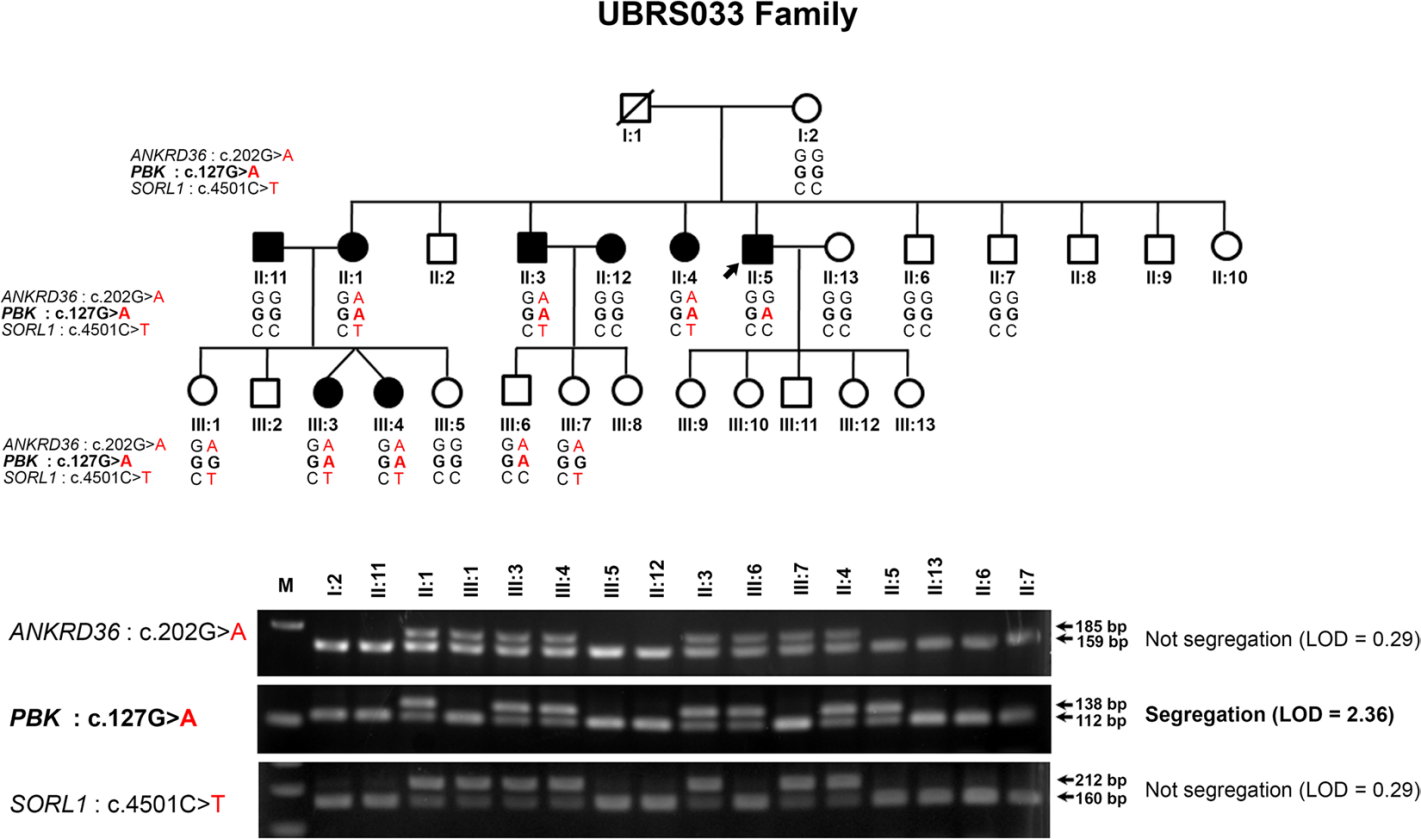
Pedigree of the UBRS033 family affected by KSD, and segregation analysis of *ANKRD36*, *PBK*, and *SORL1* variations in 16 members of the family by PCR-RFLP/dCAPS and agarose gel electrophoresis. A black circle or square represents an individual affected by KSD. Genotypes of *ANKRD36* (c.202 G > A), *PBK* (c.127 G > A) and *SORL1* (c.4501 C > T) are shown under each individual symbol. Images of the gels cropped from different gels were separated by white space. The full-length gels are presented in Supplementary Fig. S8.

To identify disease-causing genes in KSD, we selected a large family with many members (UBRS033 family) for exome sequencing and genetic study. The pedigree showed 8 affected, including a twin pair (III:3 and III:4) and 20 unaffected family members and that the KSD phenotype that was inherited as autosomal dominant model (Fig. 1). Five of affected members had opaque stones while another three had no stone detected by both KUB and ultrasound because the stone had been removed by surgery, extracorporeal shock wave lithotripsy (ESWL), or spontaneous passing. Therefore, stones from these patients were not available for the analysis of their compositions. The results of investigation for KSD by KUB radiography, clinical history, and physical examination in 16 family members are shown in Table 1. We collected blood and urine samples from proband (II:5) and his sister (II:1) (Supplementary Table S2). The serum creatinine concentrations of proband (II:5) and his sister (II:1) were 3.7 and 5.4 mg/dL, respectively, which indicated the presence of kidney failure. There were no hypercalciuria, hyperoxaluria or hyperphosphaturia presented in the proband (Supplementary Table S2).

**Table 1:** Some clinical and laboratory data of the members of the UBRS033 family.

Sample no.	Gender	Age	Age of onset*	KUB result	Site/side of stone	No. of stones/size** (cm.)	Treatment	Urine pH	Other Symptoms				Diagnosis
									Dysuria	Hematuria	Pass stone	Turbid urine	
II:11	Male	63	45	Positive/opaque stones	Renal/both	2/0.5	No	5.3	No	Yes	No	No	Renal stone (KUB^a^)
II:1	Female	59	40	Positive/opaque stones	Renal/both	1/2.0	Surgery/ESWL	5.3	No	No	No	No	Renal stone (KUB)
II:3	Male	54	43	Positive/opaque stones	Renal, ureter/both	2/2.7	No	5.8	Yes	Yes	Yes	Yes	Renal and ureteric stone (KUB)
II:4	Female	52	40	Positive/opaque stones	Renal/left	1/1.0	Surgery/ESWL	5.4	Yes	Yes	No	Yes	Renal stone (KUB)
II:12	Female	48	28	Negative			No	6.4	Yes	No	Yes	No	Renal stone (strong history^b^)
II:5	Male	48	23	Positive/opaque stones	Renal/right	2/4.0	Surgery/ESWL	5.8	No	Yes	No	No	Renal stone (KUB)
III:3	Female	34	5	Negative				5.3	Yes	Yes	Yes	No	Renal stone (strong history)
III:4	Female	34	19	Negative	Unknown/left	NA	Surgery/ESWL	5.7	No	Yes	No	Yes	Renal stone (surgical scar^c^)
I:2	Female	78		Negative				5.6	No	Yes	No	Yes	No stone
II:6	Male	47		Negative				5.9	No	No	No	No	No stone
II:7	Male	44		Negative				5.6	No	No	No	No	No stone
II:13	Female	42		Negative				5.4	No	No	No	No	No stone
III:1	Female	38		Negative				5.3	No	No	No	No	No stone
III:5	Female	29		Negative				5.7	No	No	No	No	No stone
III:6	Male	22		Negative				6.5	No	No	No	No	No stone
III:7	Female	20		Negative				6.3	No	No	No	No	No stone

ESWL = Extracorporeal shock wave lithotripsy for kidney stones.

^a^The patient had positive results of kidney–ureter–bladder (KUB) radiography. ^b^The patient had strong clinical history as justified from the presence of several symptoms associated with kidney stone, especially hematuria and stone passage. ^c^The patient had surgical scar for removal of stone.

*Age of onset of stone, **Size of the biggest stone.

### Exome sequencing and analysis of genetic variations

DNA samples from five affected members (II:1, II:3, II:4, III:3, and III:4) and three unaffected members (I:2, II:6 and II:7) from the UBRS033 family were selected for exome sequencing. The nucleotide reads were mapped against UCSC hg19 by Burrows-Wheeler Aligner (BWA), and variations (SNPs and Indels) were detected by Sequence Alignment/Map (SAMTOOLS). Genetic variations acquired from exome sequencing in these eight family members, which included 224,121 variations with an average read depth of 38×, were analyzed. Initially, the variations outside exonic regions were excluded. Since KSD in this family inherited as the autosomal dominant model, heterozygous variations that were shared among the five affected members, but absent in the unaffected members, were reserved for subsequent analysis. After filtration by exclusion of synonymous variations, but selection for non-synonymous, stop, gain/loss, short insertion or deletion (indel) variations, and rare variants (frequency <0.01), 17 variations remained for analysis in the next step (Supplementary Fig. S1 and Table 2).

**Table 2:** Prediction of the impact of amino acid changes on the protein structures and functions of 17 candidate variations by 6 web-based programs.

CHR.	POS.	Gene name	dbSNP	1000 G Frequency	Effect	Nucleotide changes	Amino acid changes	Prediction* (of 6 programs)
**2**	**97783805**	***ANKRD36***	**rs571299992**	**0.00199681**	**Missense variant**	**c.202 G** > **A**	**p.Ala68Thr**	**5**
**8**	**27685647**	***PBK***	—	—	**Missense variant**	**c.127 G** > **A**	**p.Gly43Arg**	**5**
1	85028969	*CTBS*	rs3768249	0.0091853	Missense variant	c.928 G > T	p.Asp310Tyr	3
12	77427693	*E2F7*	rs139349075	0.00159744	Missense variant	c.1253 A > G	p.Glu418Gly	3
5	156769914	*FNDC9*	rs201701167	0.000399361	Missense variant	c.631 G > T	p.Gly211Trp	3
12	10588530	*KLRC2*	rs75545535	—	Missense variant	c.56 G > C	p.Arg19Pro	3
12	110944398	*RAD9B*	rs552692137	0.000599042	Missense variant	c.288 C > G	p.Ile96Met	3
**11**	**121466463**	***SORL1***	—	—	**Missense variant**	**c.4501 C** > **T**	**p.Arg1501Trp**	**3**
8	30701641	*TEX15*	rs142485241	0.00638978	Missense variant	c.4893 G > C	p.Gln1631His	3
2	98525148	*TMEM131*	rs535701294	0.00179712	Missense variant	c.263 T > C	p.Leu88Pro	3
22	20761028	*ZNF74*	rs190749586	0.000998403	Missense variant	c.1705T > C	p.Ser569Pro	3
9	34725069	*FAM205A*	rs114933270	—	Missense variant	c.2168 A > T	p.Glu723Val	2
12	15035203	*MGP*	rs374434209	—	Missense variant	c.257 G > A	p.Arg86His	2
18	77895740	*ADNP2*	rs113879497	0.00359425	Missense variant	c.2444 A > G	p.Asn815Ser	1
5	140229086	*PCDHA9*	rs251354	—	Missense variant	c.1006 C > G	p.Leu336Val	1
2	103149147	*SLC9A4*	rs79918239	0.00179712	Stop retained variant	c.2397 G > A	p.Ter799=	1
12	123810152	*SBNO1*	rs373314152	0.000199681	Splice region variant & intron variant	c.1876-6 C > T	—	0

CHR. = chromosome, POS. = position.

*Number of programs that predict the impact of amino acid changes on the protein structures and functions as “Pathogenic”, “Damaging”, “Probably damaging”, “Possibly damaging”, “Disease causing”, or “Deleterious”.

The impact of amino acid changes on the protein structures and functions of the 17 candidate variations was predicted by 6 web-based programs, including Polymorphism Phenotyping v2 (PolyPhen-2)^30^, VarioWatch^31^, MutationTaster^32^, Sorting Intolerant From Tolerant (SIFT)^33^, Mutation Assessor^34^, and Likelihood Ratio Test (LRT)^35^. Two novel variations in *PBK* (c.127 G > A, p.Gly43Arg) and *SORL1* (c.4501 C > T, p.Arg1501Trp), and one reported variation in *ANKRD36* (rs571299992, c.202 G > A, p.Ala68Thr) were predicted to be disease-causing or damaging by 5, 3, and 5 of 6 programs, respectively (Table 2).

### Genetic analyses revealed a novel substitution in PBK as disease-associated variant

**Figure 2:**
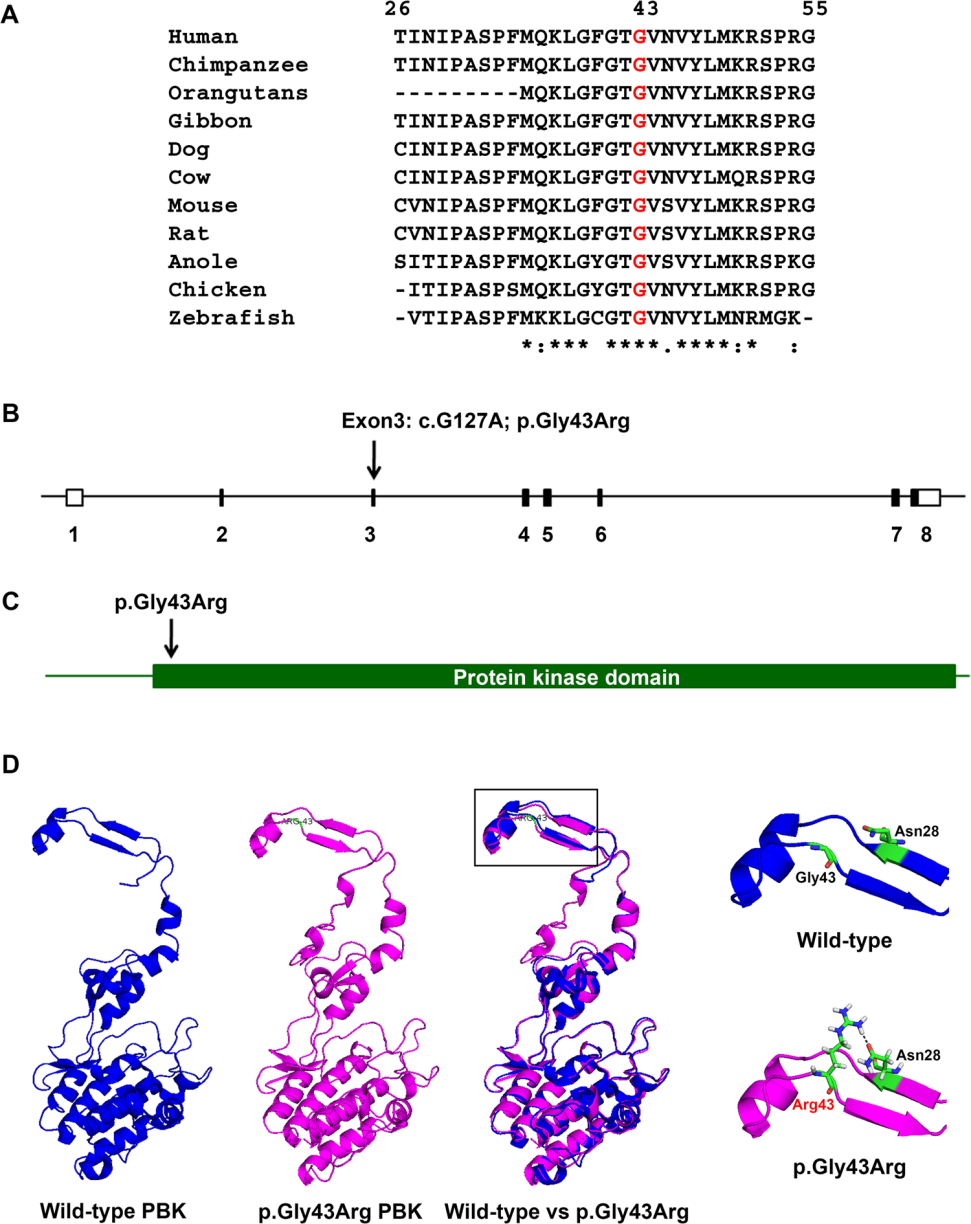
Multiple amino-acid sequence alignment, schematic diagram of the PBK structure, and three-dimensional (3D) structure of PBK protein. (**A**) Multiple amino acid sequence alignment of PBK from eleven vertebrate species in the regions where the p.Gly43Arg variation was identified. (**B**) Schematic diagram of the structure of *PBK* and the location of nucleotide variation c.G127A in exon 3 (arrow). Exons and introns are represented by boxes and lines, and exons are numbered. (**C**) Protein structure of PBK, and the location of amino acid change (p.Gly43Arg) attributable to nucleotide variation in the gene. The 322 amino acids of the PBK protein are represented by a solid line with protein kinase domain. (**D**) Three-dimensional and superimposed structures of wild-type and p.Gly43Arg PBK proteins. The structure of wild-type PBK (5J0A, blue color), p.Gly43Arg PBK (magenta color) as predicted by Swiss-model homology modeling server, and superimposed structures of wild-type and p.Gly43Arg PBK proteins (blue and magenta colors). Square box indicates the protein region where the alteration is located. There is no putative H-bond connecting to Gly43 in the wild-type structure (top-right, blue and green colors). In contrast, there is one putative H-bond connecting Asn28 to Arg43 in the p.Gly43Arg structure (bottom-right, magenta and green colors).

Three variations predicted to be pathogenic or damaging were genotyped in all affected and unaffected members of the index family. Of those, only p.Gly43Arg variation in *PBK* gene was cosegregated with KSD in the family (LOD scores = 2.36) (Table 3 and Fig. 1). One family member (III:6; aged 22 years) carried the variation without having KSD, which is possible since the disease usually develops at an age >25 years. The *PBK* p.Gly43Arg variation was not present in the DNA samples of 180 normal control subjects as screened by polymerase chain reaction and high resolution melting (PCR-HRM) analysis (Supplementary Fig. S3). Haplotype analysis suggested that the allele of *PBK* p.Gly43Arg variation was inherited from the father (I:1) who is deceased (Fig. 1 and Supplementary Fig. S2). Amino-acid sequence alignment demonstrated that Gly43 is highly conserved in the evolution of vertebrates (Fig. 2A), which supports its functional significance in the protein structure. The p.Gly43Arg variation was found to be located at exon 3 of *PBK*, and within the protein kinase domain of the PBK protein (Fig. 2B,C). PBK protein modeling and *in silico* mutagenesis indicated that the substitution (p.Gly43Arg) affects the protein structure by alterations of the H-bond forming patterns (Fig. 2D).

**Table 3:** Genotyping and segregation analyses of 3 selected variations in the UBRS033 family.

Chromosome	Gene	Variation	Segregation in UBRS033 family	LOD	Possible causative variants
2	*ANKRD36*	p.Ala68Thr	No	0.29	No
8	*PBK*	p.Gly43Arg	Yes	2.36	Yes*
11	*SORL1*	p.Arg1501Trp	No	0.29	No

*This variation was genotyped by PCR-HRM and was not present in the DNA samples of 179 subjects with KSD and 180 normal control subjects.

### Gene scanning and analysis of genetic variations

To examine whether *PBK* p.Gly43Arg variation or other variations of *PBK* were present in additional patients with KSD, we genotyped these variations and screened all 8 exons (including their exon-intron boundaries) of *PBK* in the DNA samples from 180 patients with KSD (including one sample from the index family as a positive control sample) by PCR-HRM method, followed by DNA sequencing. A total of 13 variations in *PBK* (6 novel and 7 reported variations) including p.Gly43Arg were identified (Supplementary Fig. S4 and Supplementary Table S3). All exonic variations in *PBK* were analyzed for their impact on protein structure and function using the 6 previously mentioned web-based programs, and the impact of exon-intron boundaries on mRNA splicing process was evaluated using ESEfinder 2.0^36^.

The six web-based programs revealed p.Gly43Arg to be the only variation that was predicted to affect function of the protein (Supplementary Table S3). A non-synonymous variation (c.320 A > G [rs3779620]) was observed, but it is also present in high frequency in the 1,000 Genomes Project database, so it was excluded as a disease-associated variant. We found 4 variations (c.−99C > A [rs3735744], c.14 G < C, c.295 + 4 C > A [rs727813] and c.*683_*685delAAG) that were predicted to affect exonic splicing enhancer and cryptic splice site; thus, these variations were analyzed in the 180 normal control subjects by PCR-HRM. The variations c.−99C > A, c.295 + 4 C > A, and c.*683_*685delAAG were present in 29, 88, and 90 normal control subjects, respectively (Supplementary Fig. S5). Even though the variation c.14 G < C was not present in normal control subjects and could not be tested for cosegregation with KSD in the families because only one affected individual was available for testing, the nucleotide and amino-acid sequence alignment revealed them not to be conserved in the evolution of vertebrates.

### mRNA and protein expression, immunohistochemistry, and immunofluorescence staining in human kidney tissues

**Figure 3:**
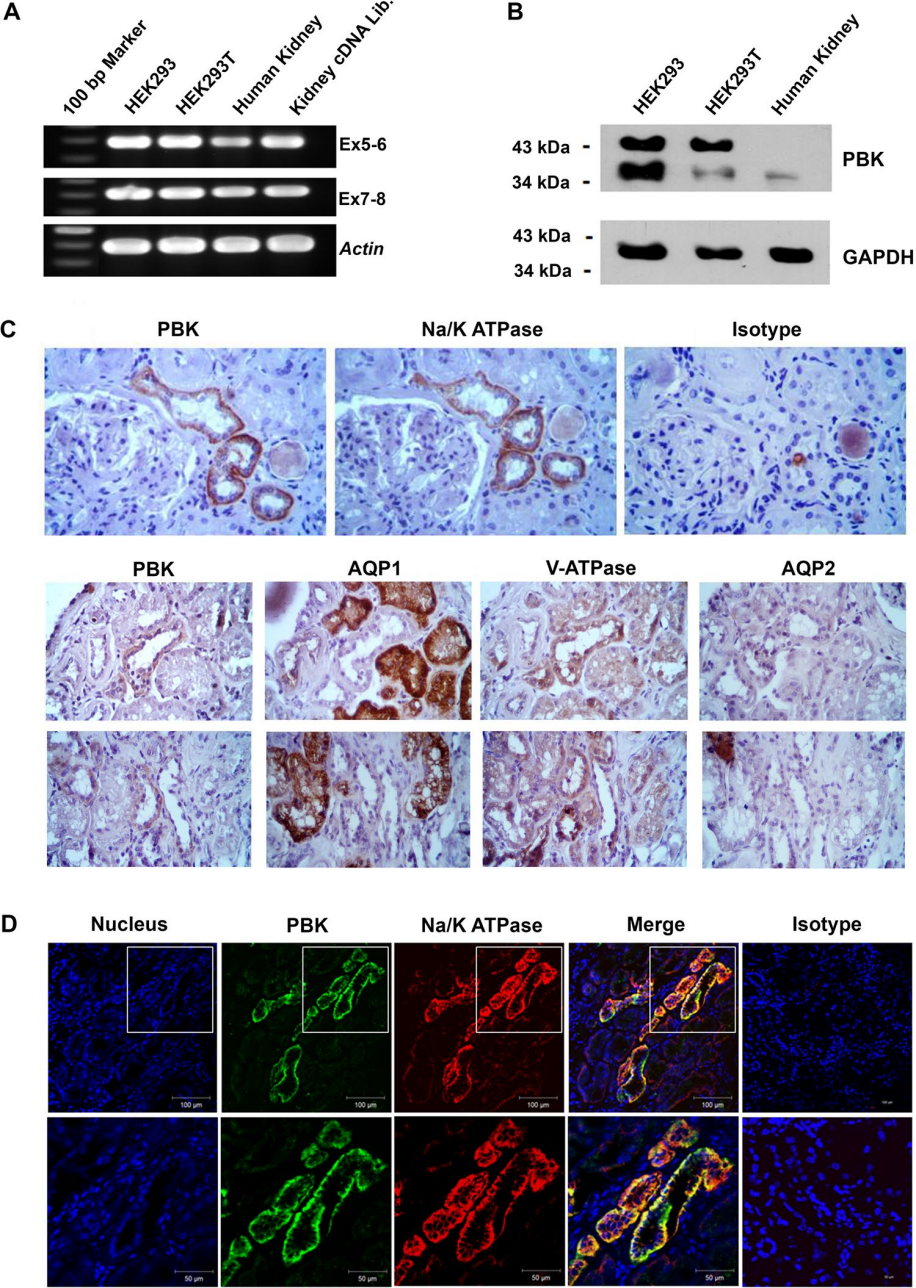
Expression of *PBK* mRNA and PBK protein in kidney cell lines and human kidney tissue. (**A**) *PBK* mRNA expression in HEK293 cells, HEK293T cells, human kidney tissue, and kidney cDNA library detected by RT-PCR method. Two regions of *PBK* mRNA covering exons 5–6 and 7–8 were analyzed, and mRNA of the house-keeping gene *ACTB* was used as an internal control. (**B**) PBK protein expression (expected size:36 kDa) in HEK293 cells, HEK293T cells, and human kidney tissue detected by immunoblot analysis. GAPDH was used as loading control. (**C**) Staining of PBK in human kidney tubules by immunohistochemistry (IHC) method using PBK-specific antibody compared to that using isotype control antibody. Na ^+^ /K^ +^ -ATPase was stained as a marker to localize tubules (Upper panel). AQP1was used as protein marker of proximal tubule, V-ATPase as protein marker of distal tubule and collecting duct, and AQP2 as protein marker of collecting duct (Two lower panels). (**D**) Staining of PBK (green) and Na ^+^ /K^+^−ATPase (red) in human kidney tubules by immunofluorescence (IFA) method showing their colocalization at the basolateral membrane in tubular cells. Blue indicates DAPI staining of nuclei. Signals were visualized by confocal microscopy (scale bars: top 100 µm, and bottom 50 µm). Images of the gels and blots cropped from different parts of the same gel/blot, or from different gels/blots were separated by white space. The full-length gels and blots are presented in Supplementary Fig. S9.

To investigate *PBK* expression in human kidney tissues, we examined its mRNA by reverse transcriptase – polymerase chain reaction (RT-PCR), and its protein by immunoblotting analysis. Figure 3A,B shows the expression of *PBK* mRNA and protein in human kidney tissues and cell lines, respectively. The expected size of PBK protein is 36 kDa, but there was an upper band (at about 43 kDa) visible in HEK293 and HEK293T cell lines. The larger size might result from post-translational modifications of PBK protein in HEK293 and HEK293T cell lines, which may not occur in human kidney tissues. In addition, we stained PBK protein in human kidney tissue sections by immunohistochemistry (IHC) and immunofluorescence (IFA) technique (Fig. 3C,D). The PBK protein is coexpressed with Na^+^/K^+^-ATPase, which is a tubular marker, indicating that PBK is expressed in tubules of nephron. Furthermore, we stained protein in human kidney tissue sections with segment specific marker proteins. PBK was not observed with AQP1 and AQP2 that are expressed in proximal tubule and collecting duct, respectively, suggesting that PBK is expressed in the distal tubule of nephrons (Fig. 3C and Supplementary Fig. S6).

### Evaluation of wild-type and p.Gly43Arg PBK proteins expressed in HEK293T cells

**Figure 4:**
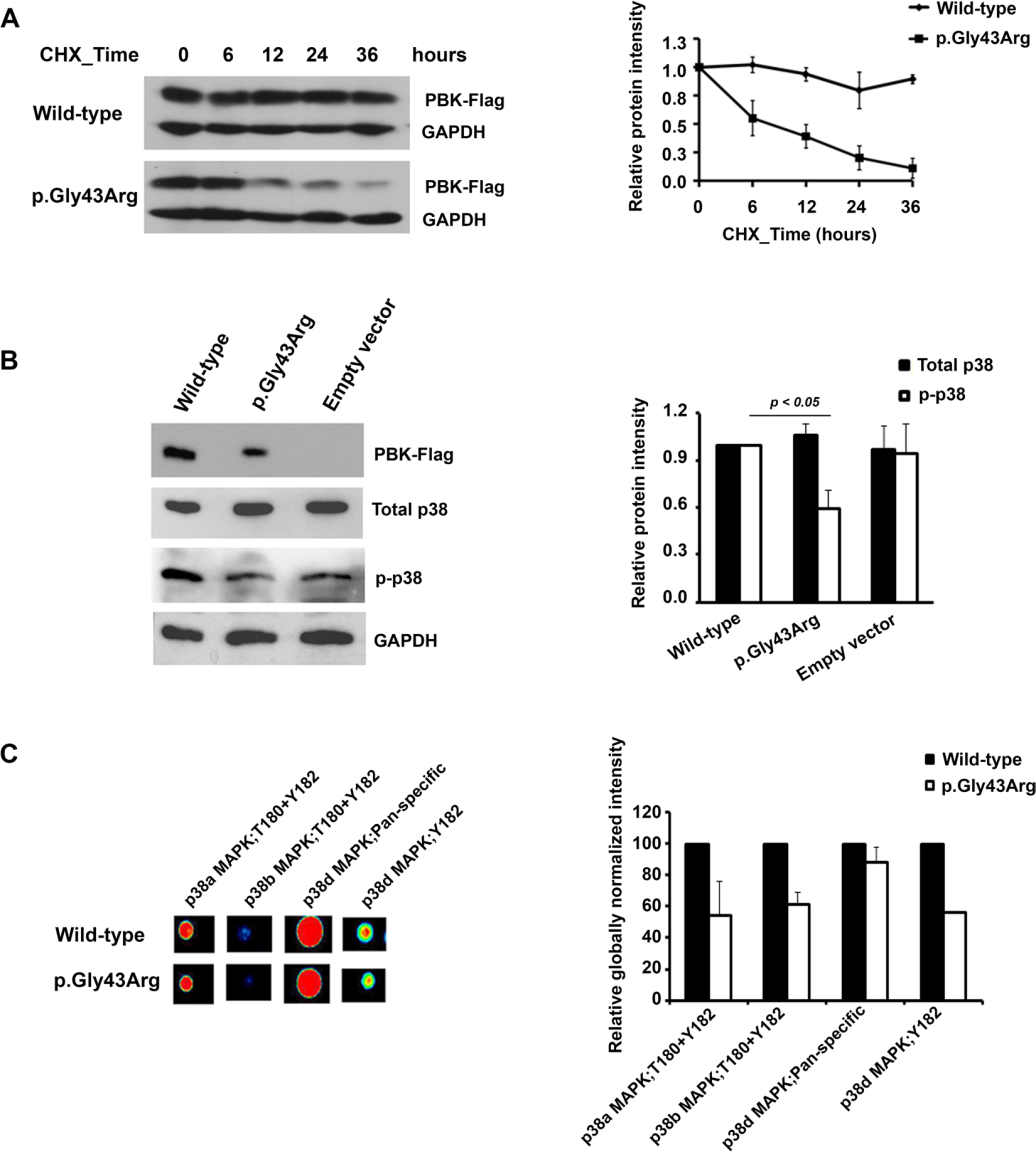
Evaluation of wild-type and p.Gly43Arg PBK expressed in HEK293T cells. (**A**) The wild-type and p.Gly43Arg PBK proteins in transfected HEK293T cells after treatment with 100 μg/ml of cycloheximide (CHX) for 0, 6, 12, 24, and 36 hours as detected by immunoblot method (left). The wild-type and p.Gly43Arg PBK proteins were quantified by ImageJ 1.50i^56^ and plotted as relative intensities. The data were shown as the mean ± SEM of three independent experiments (right). (**B**) Level of p38MAPK (total p38) and phosphorylation of p38MAPK (p-p38) in HEK293T transfected with wild-type, p.Gly43Arg, or empty vector as detected by immunoblot method (left). GAPDH was used as loading control. The bar graph shows staining densities of the proteins normalized by that of GAPDH. Results are shown as the mean ± SEM of the relative signal intensity from three independent experiments (right). (**C**) The antibody microarray analysis showed the spot intensities of the p38 MAPK family (p38-alpha, p38-beta, p38-delta) in HEK293T expressing p.Gly43Arg PBK compared to wild-type PBK (left). The bar graphs represent relative globally normalized intensity of the p38 MAPKs in HEK293T expressing p.Gly43Arg PBK compared to wild-type PBK. The results are shown as means ± % error ranges of the globally normalized intensity values in duplicate (right). Images of the blots cropped from different parts of the same blot, or from different blots were separated by white space. The full-length blots, multiple exposures, and full scanned-images of antibody microarray are presented in Supplementary Fig. S10–12.

To examine the stability of the wild-type (WT) and variant PBK (p.Gly43Arg) proteins, transient transfection of FLAG-tagged WT and p.Gly43Arg PBK were performed in HEK293T cells, and the newly synthesized proteins were inhibited with cycloheximide – a protein synthesis inhibitor. The level of WT PBK was initially constant, and then it was slightly reduced after cycloheximide treatment for more than 12 hours (Fig. 4A). In contrast, the level of variant p.Gly43Arg PBK was more rapidly reduced than that of WT PBK. The time required to achieve a 50% reduction of variant p.Gly43Arg PBK was more than 6 hours, and it seemed to demonstrate time-dependent reduction during 0–36 hours (Fig. 4A). Moreover, we investigated the effect of variant p.Gly43Arg PBK on cell proliferation and cell apoptosis. There was no difference in cell viability and cell apoptosis of HEK293T transfected with plasmid construct containing either *WT-PBK cDNA* or *mutant-p.G43R-PBK cDNA* (Supplementary Fig. S7A—C). It is possible that endogenous PBK in HEK293T might contribute to the indifferent cell viability. However, in the conditions with the presence of 500 µM H_2_O_2_, the cell viability of HEK293T transfected with plasmid construct containing *mutant-p.G43R-PBK cDNA* trended to be slightly lower than that transfected with plasmid construct containing *WT-PBK cDNA* (Supplementary Fig. S7D)

### Downstream signaling analysis

Activation of p38 MAPK was evaluated by immunoblotting analysis of HEK293T transfected with plasmid construct containing either *WT-PBK cDNA* or *mutant-p.G43R-PBK cDNA*. The results showed that phosphorylation of p38 MAPK in the HEK293T expressing variant p.Gly43Arg PBK was 40% lower than that in the HEK293T expressing WT PBK (Fig. 4B).

The antibody microarray analysis surveyed over 613 signaling components from the major established signaling pathways, and it probed both phosphorylated and total protein levels. Using HEK293T transfected with either *WT-PBK cDNA* or *mutant-p.G43R-PBK cDNA* constructs, signaling hits between cells expressing WT PBK and variant p.Gly43Arg PBK (control and treatment) for 24 hours were compared. We used the changes in globally normalized data between control and treatment samples as the percent change from control (%CFC) to filter the data points with %CFC ≥ 60, sum of % error ranges <0.85 ×%CFC value and at least one globally normalized intensity value ≥1,000. That screening generated several hits that were different between the PBK expressions (Table 4). We noted that several of these hits were signaling proteins that are implicated in cell cycle pathways (MDM2, p53, CHK1, CDK1, CDK2, CDK7, CCNE1, and ABL1). We explored the expression of the p38 MAPK family (p38-alpha, p38-beta, p38-delta) in HEK293T expressing variant p.Gly43Arg PBK compared to WT PBK. The results showed that phosphorylation of p38 MAPK in the HEK293T-expressing variant p.Gly43Arg PBK was 39–46% lower than that of p38 MAPK in the HEK293T-expressing WT PBK (Fig. 4C), and this result is consistent with that of the immunoblotting analysis.

**Table 4:** Downstream signaling hits between cells expressing wild-type PBK and cells expressing p.Gly43Arg PBK (control and treatment) for 24 hours as analyzed by KAM-900P Antibody Microarray.

Target Protein Name	Phospho Site (Human)	Full Target Protein Name	Wild-type		p.Gly43Arg		%CFC (p.Gly43Arg from Wild-type)
			Globally Normalized intensity	%Error Range	Globally Normalized intensity	%Error Range	
STAT2	Y690	Signal transducer and activator of transcription 2	12.07	25.00	3998.24	30.54	33020.71
CTNNB1	Pan-specific	Catenin (cadherin-associated protein) beta 1	8899.32	36.86	39376.07	34.98	342.46
ABL1	Pan-specific	Abelson proto-oncogene-encoded protein-tyrosine kinase	370.84	1.81	1007.12	69.04	171.57
CDK2	T160	Cyclin-dependent protein-serine kinase 2	4985.72	4.41	13344.65	89.59	167.66
CDK1	T14	Cyclin-dependent protein-serine kinase 1	994.27	14.90	2458.99	97.52	147.32
CAV2	Pan-specific	Caveolin 2	2203.31	14.29	4762.67	31.30	116.16
MKK4	Pan-specific	MAPK/ERK protein-serine kinase 4	1560.23	13.09	3060.01	8.75	96.13
BRSK1	T189	BR serine/threonine-protein kinase 1	2885.11	30.13	5491.48	25.83	90.34
BCK2L7	Pan-specific	Bcl2 homologous antagonist/killer	581.21	6.27	1025.35	18.75	76.42
MAP3K7	S439	TGF-beta-activated protein-serine kinase 1	2880.44	11.14	5061.08	54.95	75.70
SIT	Y90	Signaling threshold-regulating transmembrane adapter 1	3956.22	13.01	6810.59	16.99	72.15
CCNE1	T395	Cyclin E1	12311.81	20.41	20821.20	19.41	69.12
CHK1	S280	Checkpoint protein-serine kinase 1	6169.84	13.46	10126.81	0.50	64.13
CTNNB1	S33	Catenin (cadherin-associated protein) beta 1	17747.78	3.23	29076.53	7.81	63.83
CDK7	Pan-specific	Cyclin-dependent protein-serine kinase 7	5430.82	19.64	8884.29	6.89	63.59
BLK	Y188	B lymphoid tyrosine kinase	1865.68	7.41	3028.97	14.49	62.35
CHK1	S317	Checkpoint protein-serine kinase 1	2336.62	11.57	3734.82	23.01	59.84
WNK1	T2245	Serine/threonine-protein kinase WNK1	1205.36	47.18	481.67	0.24	−60.04
p53	Pan-specific	Tumor suppressor protein p53	1547.83	26.94	602.34	18.41	−61.09
MDM2	S166	Double minute 2	11394.35	5.37	4330.00	10.92	−62.00
p70 S6K	Pan-specific	Ribosomal protein S6 kinase beta-1	1047.28	7.61	292.15	1.45	−72.10
PDGFRa	Y754	Platelet-derived growth factor receptor kinase alpha	1927.40	21.94	527.39	17.82	−72.64

%CFC = Percent change from control, this value was calculated from the changes in globally normalized data between control and treatment.

## Discussion

Genetic variations have been reported to cause KSD^13,16,25,37–40^; however, the cause of KSD in many families is still unknown. Our group is attempting to identify the disease-causing genes of KSD in Thai families affected with this disease. In this study, using exome sequencing and genetic analysis, we discovered a novel loss-of-function variation in *PBK* (p.Gly43Arg) cosegregating with KSD in the affected family with a calculated log of odds ratio (LOD) of 2.36 (Fig. 1 and Table 3). This *PBK* (p.Gly43Arg) variation was observed in only one family, and there was no evidence of its presence in 180 normal control individuals (Supplementary Fig. S3) who live in the same geographical region. This or other pathogenic variations in all 8 exons of *PBK* was/were not detected in DNA samples from a group of 179 patients with KSD as examined by PCR-HRM and sequencing methods (Supplementary Table S3). Although the variations (c.−99C > A, c.14 G < C, c.295 + 4 C > A and c.*683_*685delAAG) were predicted to affect exonic splicing enhancer and cryptic splice site, they were present in normal control subjects (Supplementary Fig. S5), or the nucleotide and amino-acid sequence alignment demonstrated that they are not conserved in the evolution of vertebrates. Thus, it is unlikely that these variations would be pathogenic.

The p.Gly43Arg substitution in *PBK* identified in the affected family with KSD is most likely a disease-associated variation because it cosegregated with the disease in 6 affected members, but it was absent in 6 unaffected members. However, one member of this family (III:6) who was 22-years-old carried the p.Gly43Arg variation without having KSD (Fig. 1). This is possible because KSD usually develops in individuals aged older than 25 years. This family member is being followed-up to observe whether KSD occurs in this case or not. Additionally, other family members in generation III should be regularly screened for asymptomatic stones and informed for the risk of having the disease. The finding of genetic factor contributing in the family may be useful to identify the target for prevention and treatment. Since the two family members, III:3 and III:4, were rather young at first kidney stone manifestation, we searched for additional variations that might modify the disease from their exome sequencing data in comparison with the data from other affected members. A set of 175 candidate genes reported to be involved in KSD, gathered from public databases and candidate gene association study databases, were evaluated for their variations associated with KSD. We found 26 variations in 19 genes that match with this set of genes (Supplementary Table S4). Only p.Ala996Ser variation in *CASR* was reported to be associated with serum calcium level and KSD^41^. However, the levels of serum calcium in these two family members were not measured; at this stage it is not known whether p.Ala996Ser variation in *CASR* would be a modifying variation in these two members or not. Although most of the variations are not reported to be involved in KSD, these variants and environmental factors may be involved in the pathogenesis of early onset of KSD. The affected II:11 and II:12 members, who married into the family, did not carry the p.Gly43Arg variation of *PBK*. It is possible that they had different genetic variations or some different type of KSD. However, in the calculation of LOD score and the analysis to identify the disease-causing gene, we did not take these two members (II:11 and II:12) into the calculation and analysis as the founding parents. Thus, they would not affect the overall genetic analysis to identify the disease-causing gene in our study.

PBK or T-LAK cell-originated protein kinase (TOPK) is a 322 amino-acid MAPKK-like serine/threonine protein kinase that was identified as an interleukin-2-induced gene in T-lymphokine-activated killer cells, and as an interaction partner with the human tumor suppressor hDlg^27^. Multiple studies have found that PBK is overexpressed in tumor and cell lines^27,29,42–44^. PBK expression is most abundant in the placenta, also present in heart muscle and the pancreas, and present at low levels in skeletal muscle, kidney, liver, and lung^27^, but there was no report on the PBK protein expression in the adult human kidney and the human protein atlas showed PBK as not expressed in the tubules. While, in our study, we found low level of PBK protein expression in distal tubules of nephron in the human kidney tissue as examined by immunohistochemistry and immunofluorescence staining techniques (Fig. 3C,D). One simple explanation is that the level of PBK protein expression in distal tubules is low and there might be variable in sensitivities of PBK antibodies used to detect this protein in human kidney tissues in different laboratories. PBK function may be important for cell cycle regulation since it is phosphorylated in a cell cycle-dependent manner at mitosis^29,45^. Moreover, phosphorylation of p38 MAPK by PBK was also reported to mediate cell survival^26,29^.

In this study, phosphorylation of p38 MAPK was evaluated by immunoblot analysis and antibody microarray. The results showed that phosphorylation of p38 MAPK in the HEK293T-expressing p.Gly43Arg PBK is 40% lower than that in the HEK293T-expressing wild-type (WT) PBK (Fig. 4B). A number of previous studies have indicated that p38 MAPK plays an important role as a mediator of apoptosis in response to stress stimuli, such as oxidative stress^46,47^. Conversely, p38 MAPK has a prosurvival function by upregulating antioxidant gene expression and preventing a high accumulation of reactive oxygen species (ROS) upon exposure to low or moderate doses of H_2_O_2_^48^. Renal tubular epithelial cells with overproduction of ROS is attributable to oxidative stress under conditions of high oxalate stimulation, which triggers epithelial cell injury, inflammation, and cell apoptosis^49,50^. We propose that p38 MAPK activation in renal tubular epithelial cells might upregulate antioxidant genes to prevent oxidative stress, cell injury, inflammation, and cell apoptosis, which are initial components of the mechanism of stone formation.

We found that in the inhibition of newly synthesized proteins by cycloheximide, the variant p.Gly43Arg PBK was more rapidly decreased than that of the WT PBK (Fig. 4A), which suggests instability of the variant p.Gly43Arg PBK that results from amino acid changes. The variant p.Gly43Arg PBK might also impair cell viability from oxidative stress (Supplementary Fig. S7D). Previously, gene knockdown of PBK led to decreased viability and increased apoptosis^29,51^. The instability of the variant p.Gly43Arg PBK observed in this study might decrease renal tubular epithelial cell viability and increase apoptosis under the oxidative stress, causing an initial risk of crystal deposition and kidney stone formation. Moreover, PBK could play a role as an AR (androgen receptor)-regulated protein^43^. There is an evidence that AR could directly upregulate hepatic glycolate oxidase and kidney epithelial NADPH oxidase subunit p22-PHOX expression. This regulation might then increase oxalate biosynthesis and oxidative stress that results in CaOx crystal formation and retention^52^. However, the roles of WT PBK and variant p.Gly43Arg PBK related to AR regulation on oxalate biosynthesis and oxidative stress through the controls of hepatic glycolate oxidase and NADPH oxidase subunit p22-PHOX expression are still unknown. 

Although no kidney stones from this family were available for chemical analysis, they were opaque stones as detected by radiography (Table 1), and the opaque stones from patients in this region of Thailand were generally found to consist primarily of calcium oxalate^17^. The risk factor for calcium oxalate stone is oxidative stress and inflammation in the condition of high oxalate stimulation. Exposure of renal epithelial cells to high oxalate levels and CaOx crystals leads to the production of ROS, development of oxidative stress, and cellular injury^49,50^. As found in this study, the instability of the variant p.Gly43Arg PBK may not allow cell survival from oxidative stress or may affect AR signaling, resulting in renal epithelial injury. The apoptosis of renal epithelial cells probably plays an important role in kidney stone formation via apoptotic cell surface, which has clusters of phosphatidylserine that can attract calcium and act as sites for the attachment of CaOx crystals^53^. The mechanism involving retention of these crystals inside the kidney or renal collecting system and further aggregation and/or secondary nucleation ultimately results in the formation of a kidney stone^5,7^.

The p.Gly43Arg variation of *PBK* was novel and rare. This alteration and other pathogenic variations of *PBK* were not found by our genotyping in the 179 unrelated kidney stone patients (Supplementary Fig. S3). However, genotyping in a larger number of patients from different populations may help to validate this result. Furthermore, while the mechanism of tubular epithelial cell injury responded to ROS is important, the roles of CaOx-induced ROS and inflammatory responses are of interest to be studied. Also, urinary biomarkers such as 8-hydroxydeoxyguanosine levels combined with metabolic profile may be used for identifying people at risk of kidney stone development^54^.

In conclusion, we discovered a novel loss-of-function variation (p.Gly43Arg) in *PBK* associated with KSD in a Thai family with several affected and unaffected members. The unstable variant p.Gly43Arg PBK reduces phosphorylation of p38 MAPK that regulates the downstream signaling pathway, including cell viability and apoptosis. The instability of the variant p.Gly43Arg PBK may affect cell survival from oxidative stress, resulting in renal epithelial injury and kidney stone formation. Further studies on the roles of wild-type and variant p.Gly43Arg or knock-out PBK in animal models to investigate the impact of tubular epithelial cell injury upon high oxidative stress and exposure to high oxalate and crystals of CaOx may elucidate the molecular mechanism of KSD associated with the PBK variation.

## Methods

### Ethics approval

This study was approved by the Human Research Ethics Committee of the Siriraj Institutional Review Board (SIRB), Faculty of Medicine Siriraj Hospital, Mahidol University, Bangkok, Thailand (COA no. Si 392/2012, and COA no. Si 133/2015). Written informed consent was obtained from all subjects before conducting the study. All methods were performed in accordance with the relevant guidelines and regulations.

### Subjects and clinical study

This part was similarly conducted in our previous projects^25^. Two hundred and fifty-six patients with kidney stone disease (KSD) and their family members were recruited at Sappasitthiprasong Hospital in Ubon Ratchathani Province, which is located in Northeastern Thailand, during 2004–2006. Patient and family demographic and clinical data were collected. Blood and urine samples were also collected. All patients and relatives were investigated for kidney stones by radiography of kidneys, ureters, and bladder (plain KUB). In suspicious cases, ultrasonography was also performed. Diagnosis of kidney stone was made based on radiography of KUB, ultrasonography, surgical scar with medical record of kidney stone operation, and/or clinical history of kidney stone and associated symptoms (i.e., back and abdominal pain, hematuria, and stone passage).

The unrelated normal control subjects for this study (n = 180) were recruited from local villages in the same areas where the KSD patients live. All control subjects were radiographically examined similar to the protocol used in KSD patients and family to ensure no presence of KSD. Gender and age data of the normal control subjects are shown in Supplementary Table S1. Genomic DNA samples from patients and control subjects were extracted from peripheral blood cells using standard phenol-chloroform method.

### Human kidney tissues and cell lines

The use of human kidney tissues in this study was approved by the Human Research Ethics Committee of the Siriraj Institutional Review Board, Faculty of Medicine Siriraj Hospital, Mahidol University. Human fresh frozen tissues from patients without KSD were obtained from remaining specimens from routine pathological examinations. Human kidney tissues, HEK293 cell line, and HEK293T cell line were used for the studies of *PBK* mRNA expression by reverse transcription and polymerase chain reaction (RT-PCR) method. The encoded PBK protein expression was determined by immunoblot analysis, double immunofluorescence staining, and immunohistochemistry staining.

### Genetic analysis

Exome sequencing and data analysis was performed in a selected family (UBRS033) with a maximal ELOD of 2.66 that includes 28 members (8 affected and 20 unaffected) and an affected twin. DNA samples from five affected members (II:1, II:3, II:4, III:3 and III:4) and three unaffected members (I:2, II:6 and II:7) were sent to Macrogen (Seoul, South Korea) for exome sequencing analysis. Detailed methods are given in the Supplementary Methods section.

Nucleotide sequences of genes of interest were acquired from the GenBank database to facilitate the design of polymerase chain reaction (PCR) primers (Supplementary Table S5). Exons of the genes of interest were amplified from DNA samples from KSD patients and normal control subjects using specific primer-pairs (Supplementary Table S6), and then they were genotyped and screened for genetic variations (more details are provided in the Supplementary Methods section).

### Protein structure modeling

The crystal structure of PDZ-binding kinase (5J0A) was used to serve as the wild-type PBK structure, and to generate the substitution protein structure (p.Gly43Arg) using the Swiss-model homology modeling server^55^. Alterations in the H-bond-forming pattern caused by amino acid variation were examined using PyMOL 1.7.5.0 (DeLano Scientific LLC, Palo Alto, California, USA).

### Gene expression in human kidney, and transient transfection in HEK293T cells

Total RNA was extracted from human fresh frozen kidney tissues, HEK293 cells, and HEK293T cells as previously described by Nettuwakul *et al*.^25^. *PBK* cDNA was examined by RT-PCR. The primer sequences are shown in Supplementary Table S7. Immunoblot analysis of proteins extracted from human kidney tissues, and immunohistochemistry, double immunofluorescence staining, and the stability of wild-type and variant p.Gly43Arg PBK proteins expressed in HEK293T cells were performed as described in the Supplementary Methods section.

### Examination of protein phosphorylation by antibody microarray

Protein phosphorylation in HEK293T cells transfected with plasmid construct containing either *WT-PBK cDNA* or *mutant-p.G43R-PBK cDNA* was examined by KAM-900P Antibody Microarray Service (Kinexus Bioinformatics Corporation, Vancouver, Canada). The KAM-900P Antibody Microarray features 613 phosphosite-specific antibodies (for phosphorylation) and 265 pan-specific antibodies (for expression levels of these phosphoproteins) in duplicate using two samples on the same microarray slide. The transfected cells were harvested with chemical cleavage buffer according to standard Kinexus recommendations and then shipped to Kinexus Bioinformatics Corporation. The globally normalized data from Kinexus was further filtered to remove data points with error ranges, and the changes in spot intensity between control and treatment samples represent the percent change from control (%CFC).

### Statistics

Data sets were collected from at least three independent experiments, and values are expressed as mean ± SEM (standard error of the mean). One sample *t*-test was used to identify significant differences between the means of control and test. Differences with a *p*-value <0.05 were considered statistically significant.

## Supplementary information

Supplementary information.

## Data Availability

The datasets generated during and/or analyzed during the current study are available from the corresponding authors on reasonable request.
