# Supplementary material for: A novel loss-of-function mutation of PBK associated with human kidney stone disease
Source: Sci Rep. 2020 Jun 24;10:10282. doi: 10.1038/s41598-020-66936-4 (PMC7314804; doi:10.1038/s41598-020-66936-4)
Supplement: Supplementary file 1 — Supplementary information. [file 41598_2020_66936_MOESM1_ESM.pdf]

Supplementary Information

A novel loss-of-function mutation of *PBK* associated with human kidney stone disease

Choochai Nettuwakul[1], Nunghathai Sawasdee[1], Oranud Praditsap[2], Nanyawan Rungroj[2], Arnat Pasena[1], Thanyaporn Dechtawewat[1], Nipaporn Deejai[1], Suchai Sritippayawan[3], Santi Rojsatapong[4], Wipada Chaowagul[4], and Pa-thai Yenchitsomanus[1,*]

[1] Division of Molecular Medicine, Research Department, Faculty of Medicine Siriraj Hospital, Mahidol University, Bangkok, Thailand

[2] Division of Medical Genetics Research and Laboratory, Research Department, Faculty of Medicine Siriraj Hospital, Mahidol University, Bangkok, Thailand

[3] Division of Nephrology, Department of Medicine, Faculty of Medicine Siriraj Hospital, Mahidol University, Bangkok, Thailand

[4] Sappasithiprasong Hospital, Ubon Ratchathani, Thailand

***Correspondence:** Prof. Dr. Pa-thai Yenchitsomanus, Division of Molecular Medicine, Research Department, Faculty of Medicine Siriraj Hospital, Mahidol University, Bangkok 10700, Thailand.
E-mail: pathai.yen@mahidol.edu or ptyench@gmail.com

Supplementary figures and legends

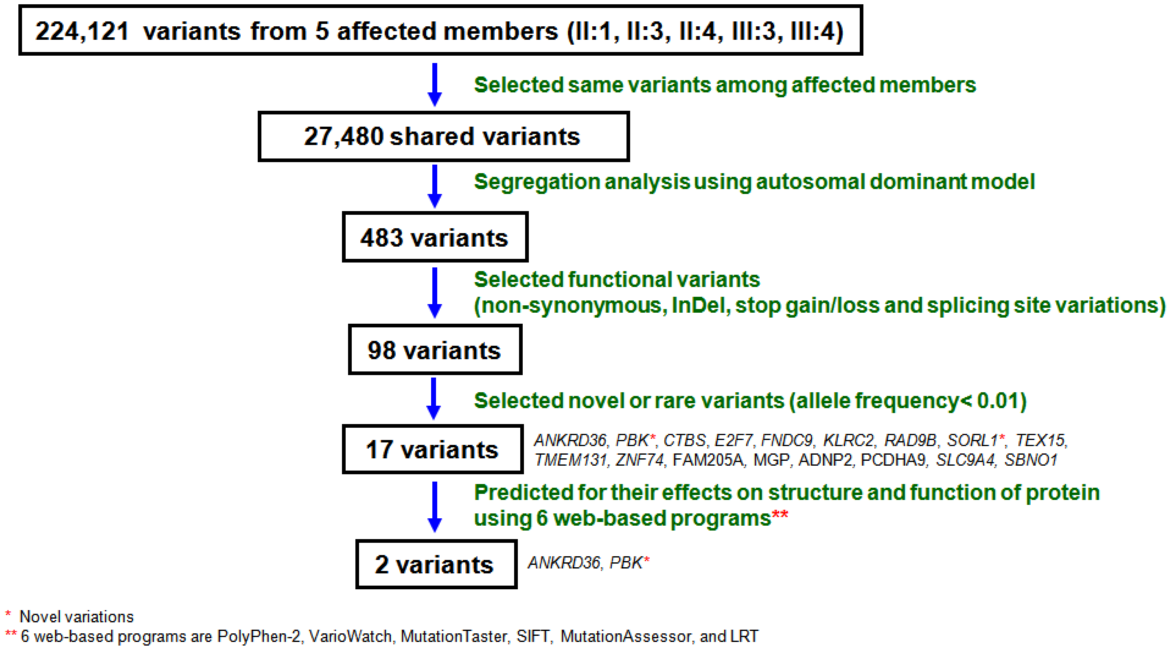

Figure S1. Filtering of genetic variations obtained from exome sequencing in a family affected with KSD.

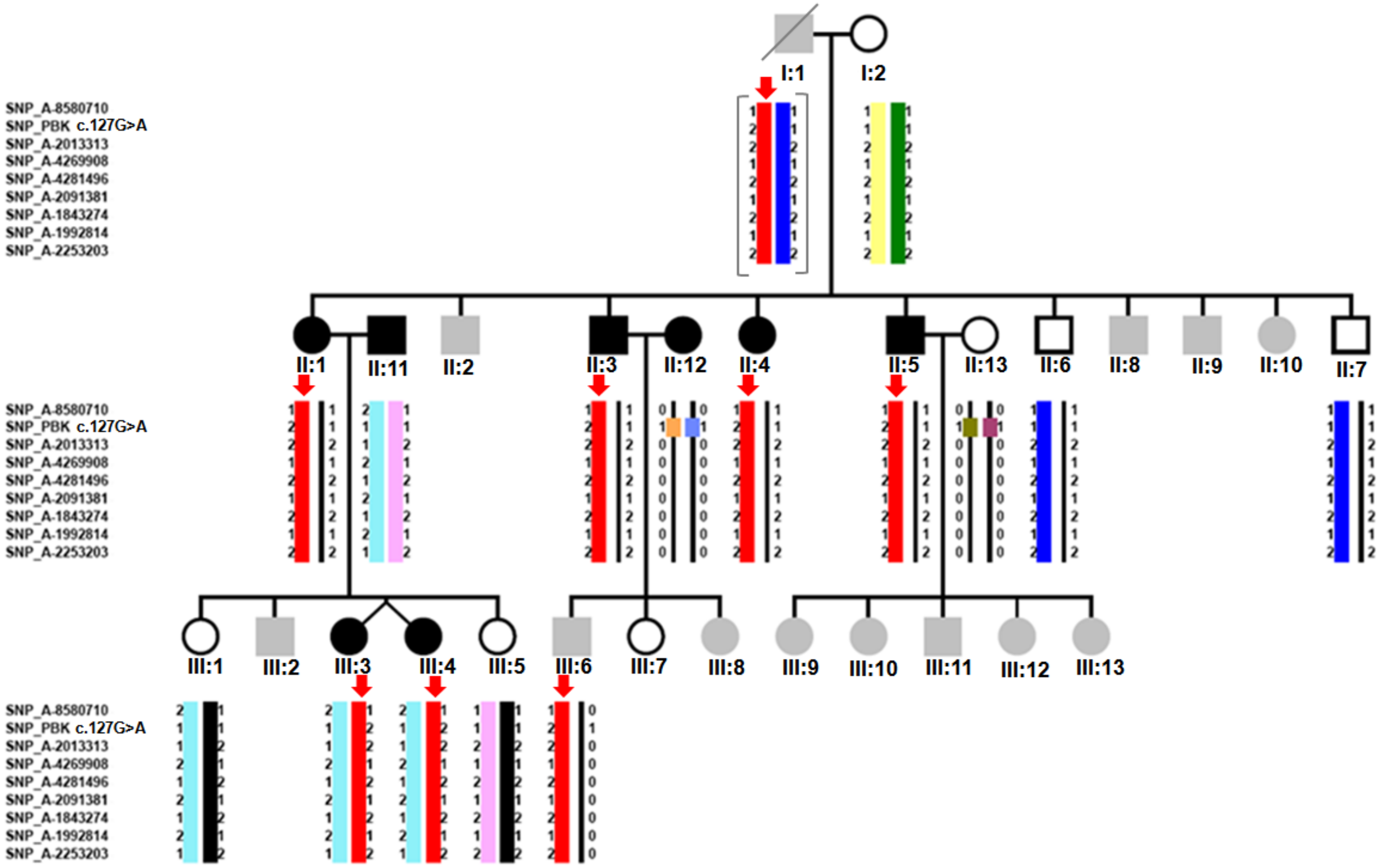

Figure S2. Haplotype analysis in the region of chromosome 8 where a novel variation in *PBK* (c.127G>A, p.Gly43Arg) and 8 single nucleotide polymorphisms (SNPs) were located, showing cosegregation of the haplotype in red color with KSD in the UBRS033 family.

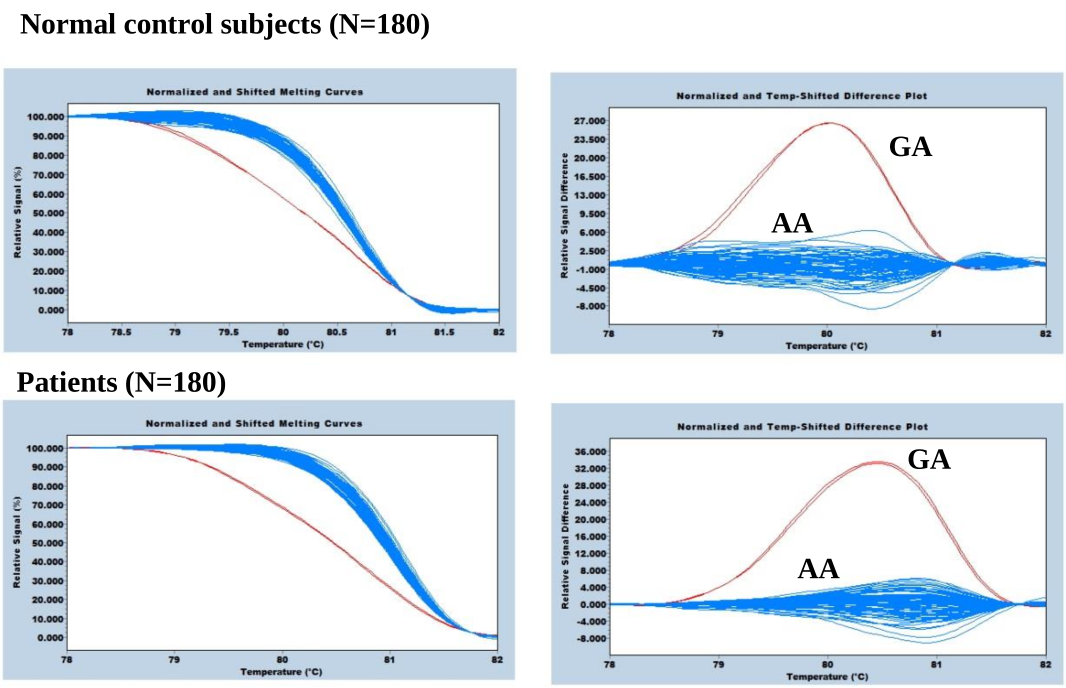

Figure S3. Genotyping of *PBK* (c.127G>A, p.Gly43Arg) in DNA samples from 180 patients with KSD, and from 180 normal control subjects by PCR-HRM method, showing melting curves and difference plots of amplicons from samples with either homozygous wild-types or heterozygous variants.

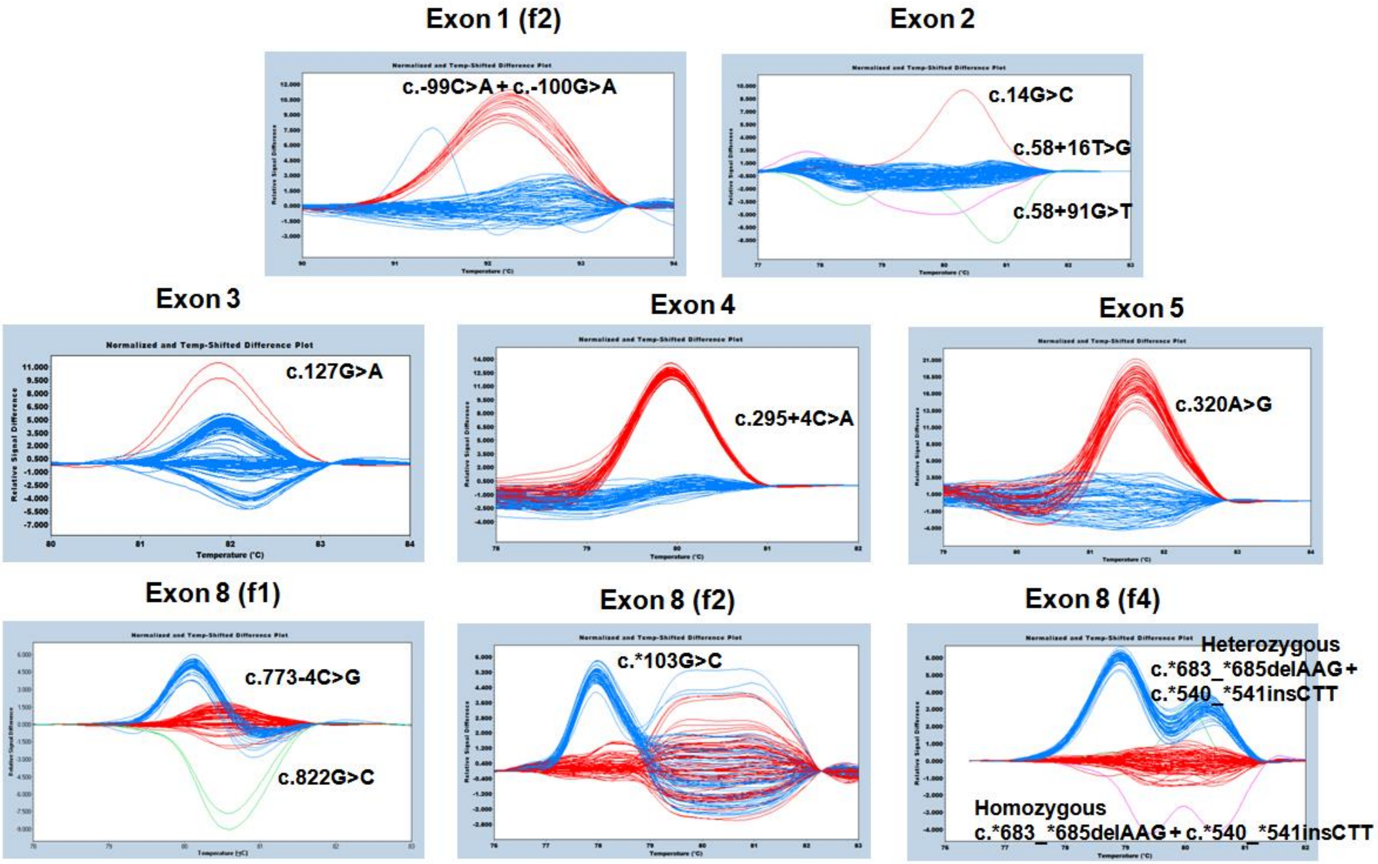

Figure S4. Screening of all 8 exons (including their exon-intron boundaries) of *PBK* in DNA samples from 180 patients with KSD by PCR-HRM method. Thirteen variations (6 novel and 7 reported variations) were identified, which were subsequently confirmed by Sanger DNA sequencing method.

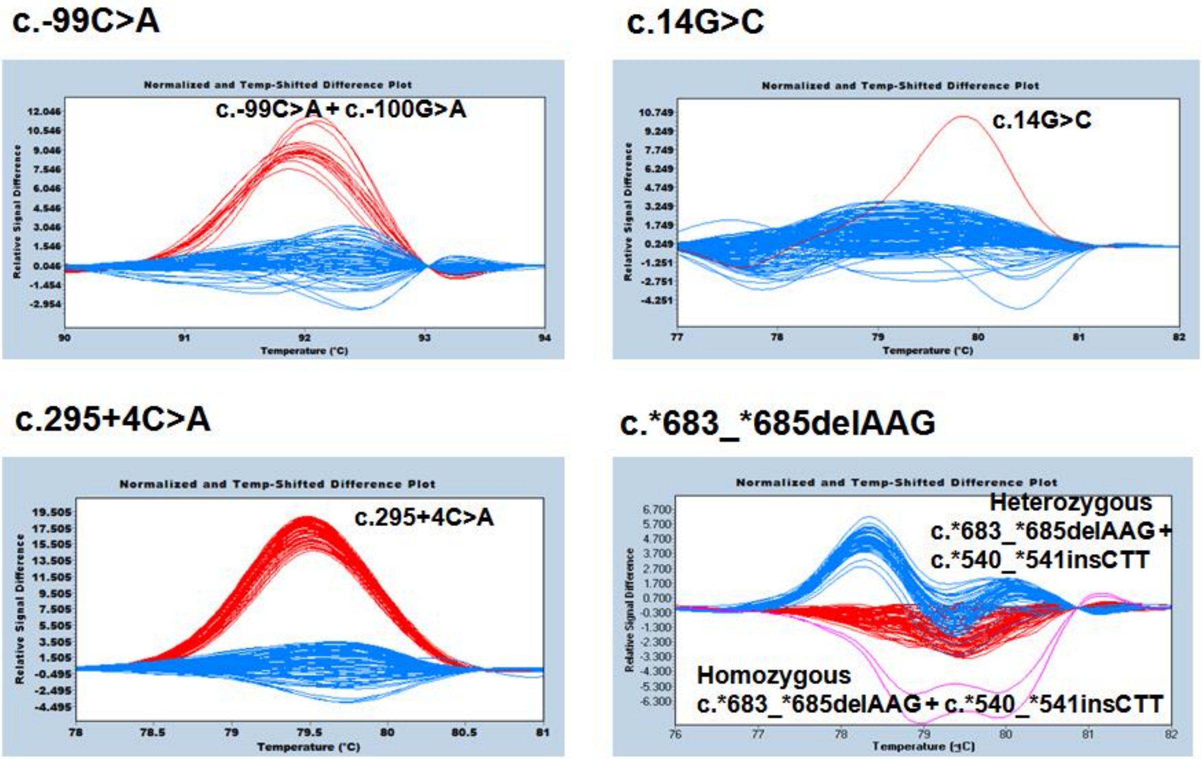

Figure S5. Genotyping of *PBK* c.-99C>A [rs3735744], c.14G<C, c.295+4C>A [rs727813], and c.*683_*685delAAG in the 180 normal control subjects by PCR-HRM method.

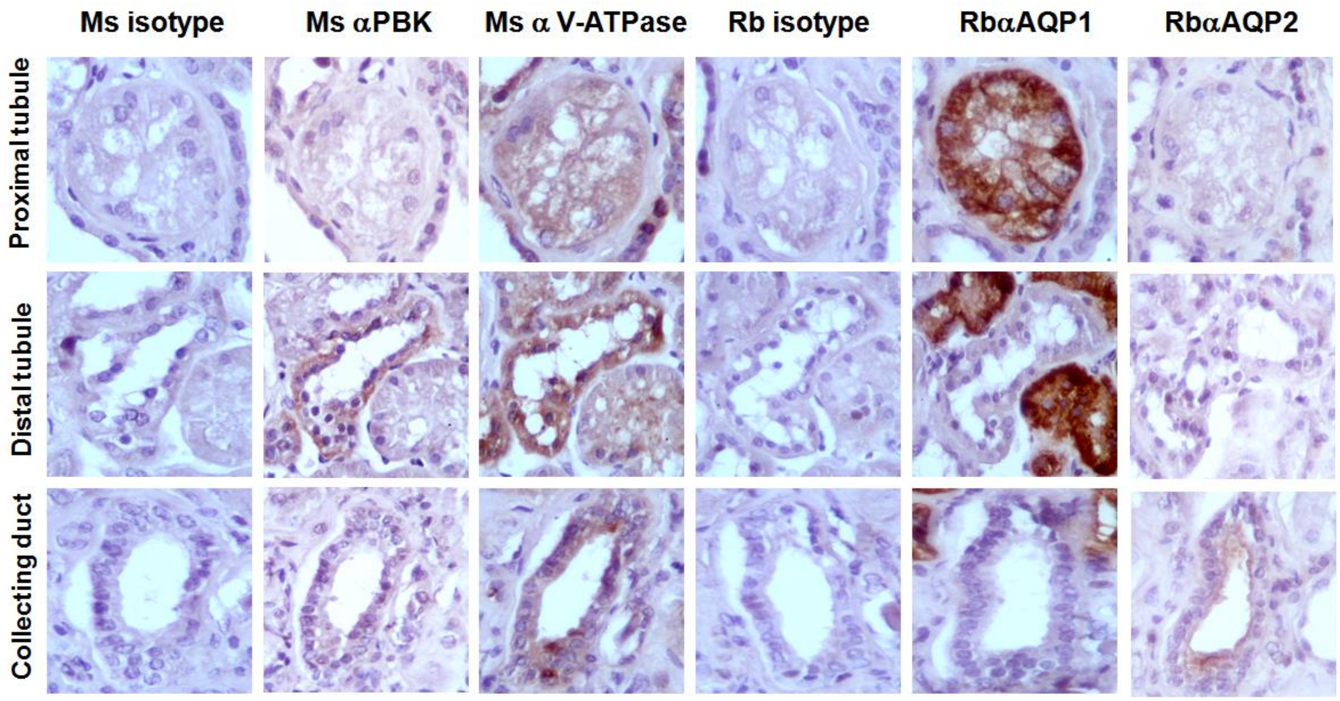

Figure S6. Detection of PBK protein by immunohistochemistry. The protein was stained in distal tubules (middle panels in the second column) of human kidney. AQP1 (fifth column) was used as protein marker of proximal tubule, V-ATPase (third column) as protein marker of distal tubule and collecting duct, and AQP2 (last column) as protein marker of collecting duct. Mouse and rabbit antibodies were used as isotype controls (first and fourth column). The original magnification was 40x.

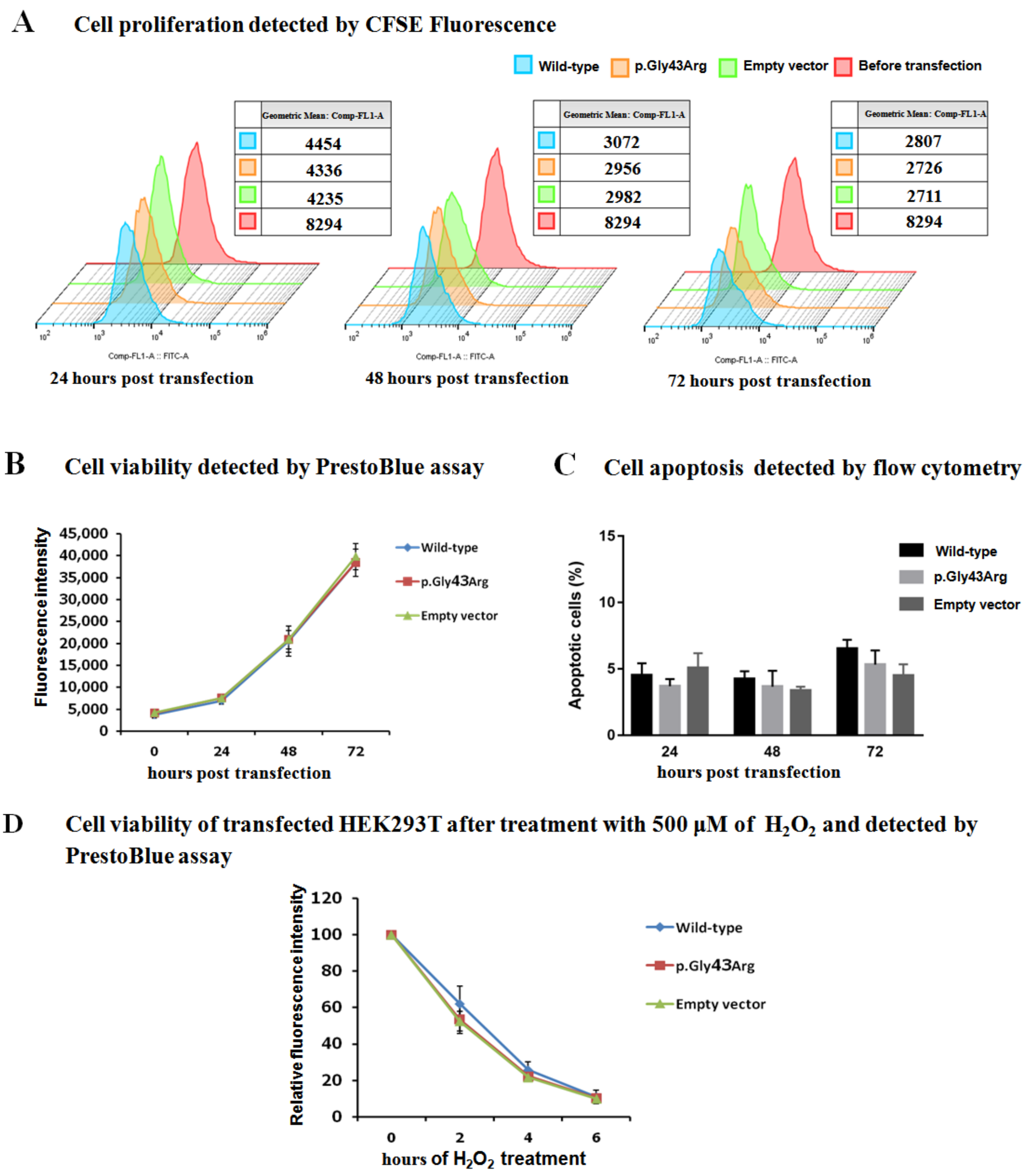

Figure S7. Cell proliferation and cell apoptosis of HEK293T transfected with wild-type, p.Gly43Arg, or empty vector . (A-B) Cell proliferation and cell viability of HEK293T transfected with wild-type, p.Gly43Arg, or empty vector as detected by (A) CFSE Fluorescence and (B) PrestoBlue assay. (C) Cell apoptosis of HEK293T transfected with wild-type, p.Gly43Arg or empty vector as detected by Annexin

V-APC/PI staining and flow cytometry. (D) Cell viability of HEK293T transfected with wild-type, p.Gly43Arg, or empty vector after treatment with 500 μM of H_2O_2 for 0, 2, 4, and 6 hours as detected by Pressto Blue assay.

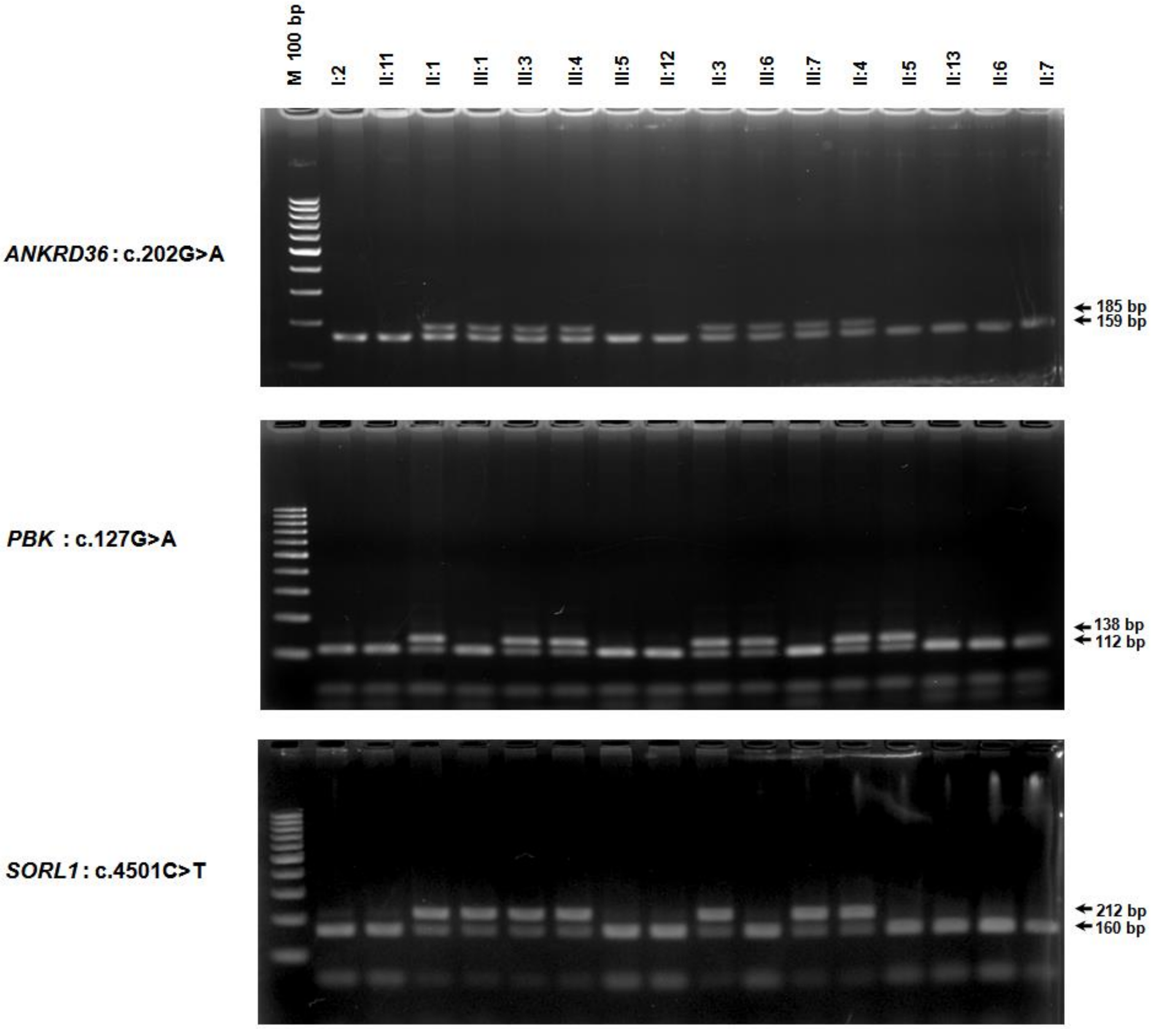

Figure S8. Segregation analysis of *ANKRD36, PBK,* and *SORL1* variations in 16 members of the UBRS033 family by PCR-RFLP/dCAPS and agarose gel electrophoresis.

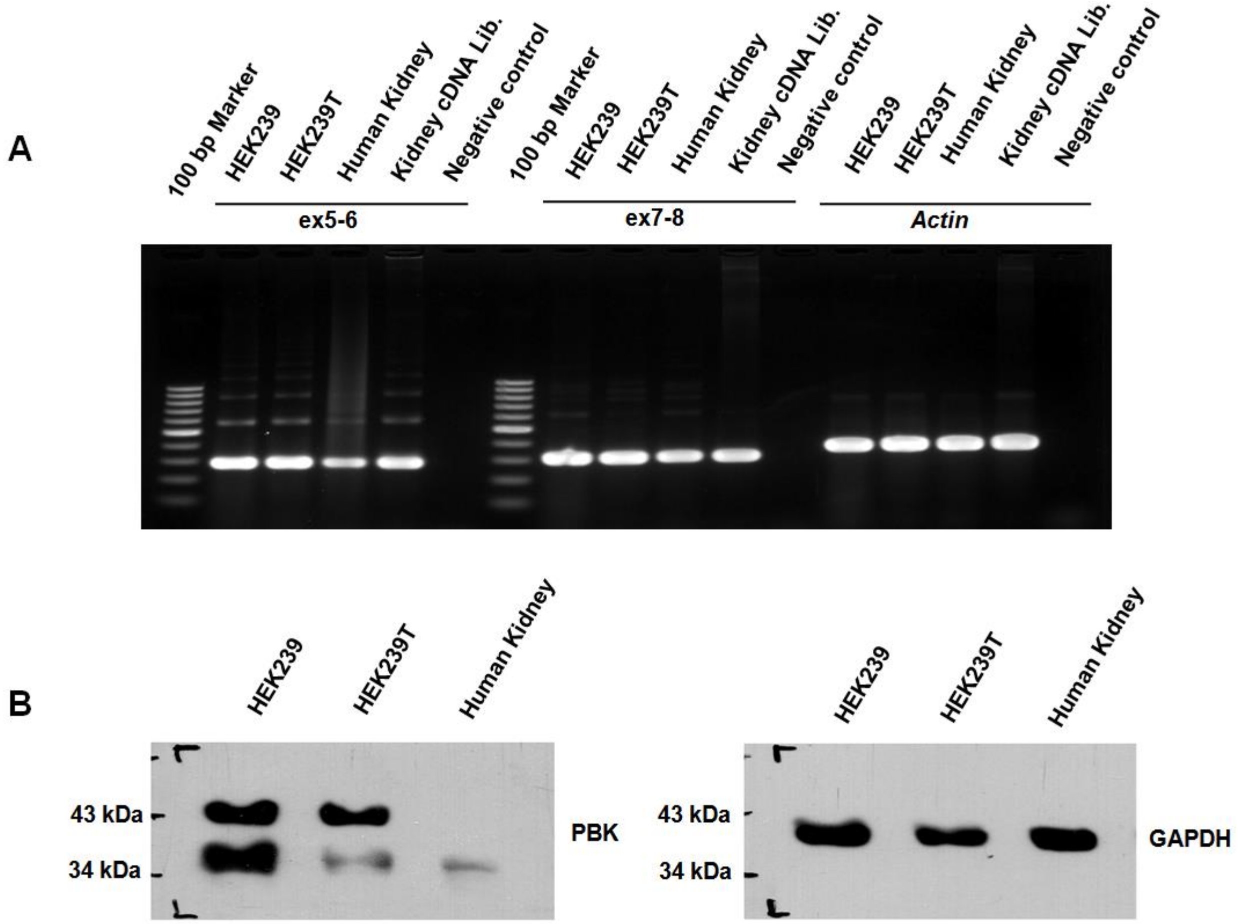

Figure S9. Expression of *PBK* mRNA and PBK protein in kidney cell lines and human kidney tissue. (A) *PBK* mRNA expression in HEK293 cells, HEK293T cells, human kidney tissue, and kidney cDNA library detected by RT-PCR method. Two regions of *PBK* mRNA covering exons 5-6 and 7-8 were analyzed, and mRNA of the house-keeping gene *ACTB* was used as an internal control. (B) PBK protein expression (expected size:36 kDa) in HEK293 cells, HEK293T cells, and human kidney tissue detected by immunoblot analysis. GAPDH was used as loading control.

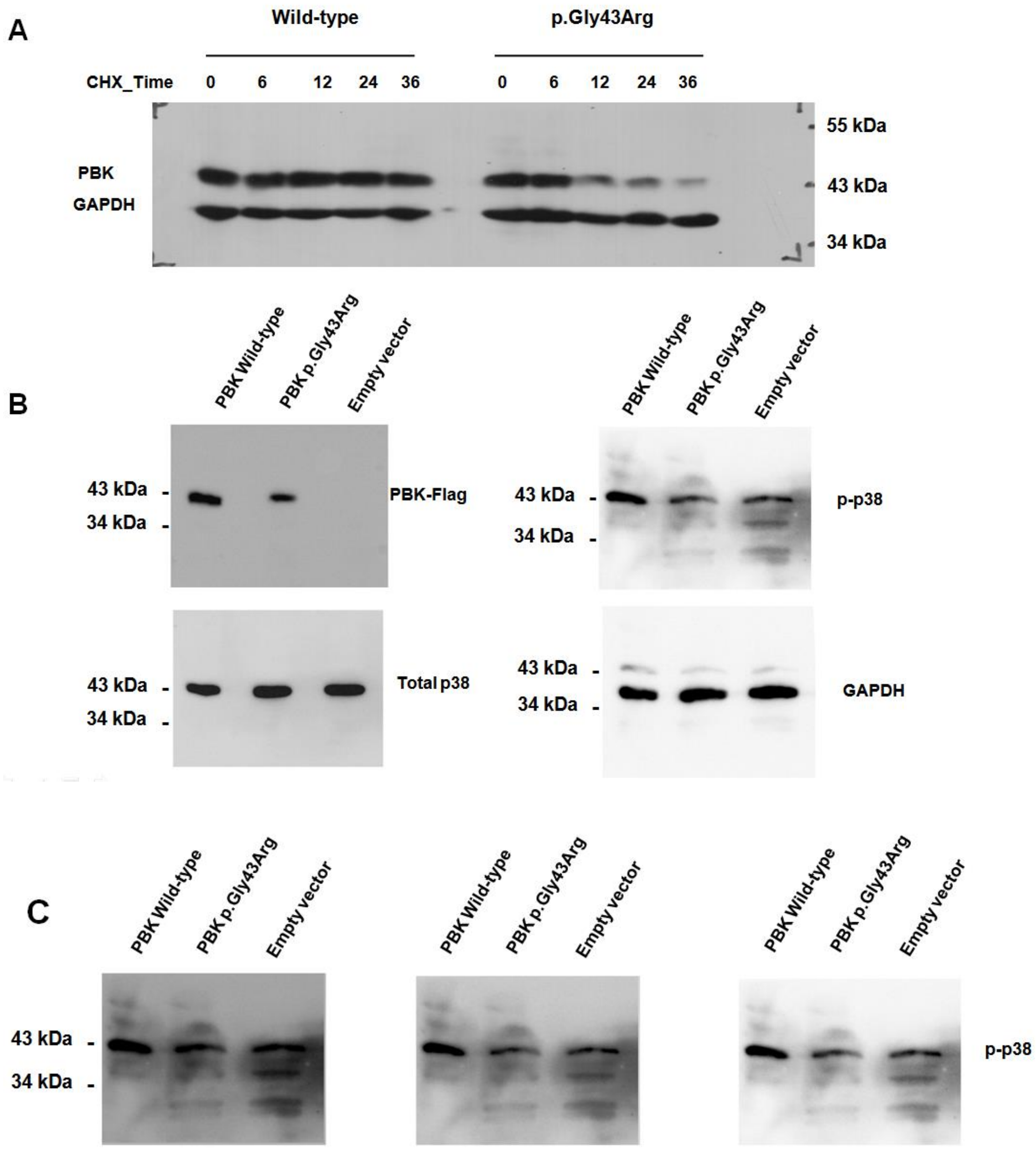

Figure S10. Stability of wild-type and p.Gly43Arg PBK expressed in HEK293T cells. (A) The wild-type and p.Gly43Arg PBK proteins in transfected HEK293T cells after treatment with 100 μg/ml of cycloheximide (CHX) for 0, 6, 12, 24, and 36 hours as detected by immunoblot method. (B) Level of p38MAPK (total p38) and phosphorylation of p38MAPK (p-p38) in HEK293T transfected with wild-type, p.Gly43Arg, or empty vector as detected by immunoblot method. GAPDH was used as loading control. (C) Multiple exposures of phosphorylated p38MAPK (p-p38) detection.

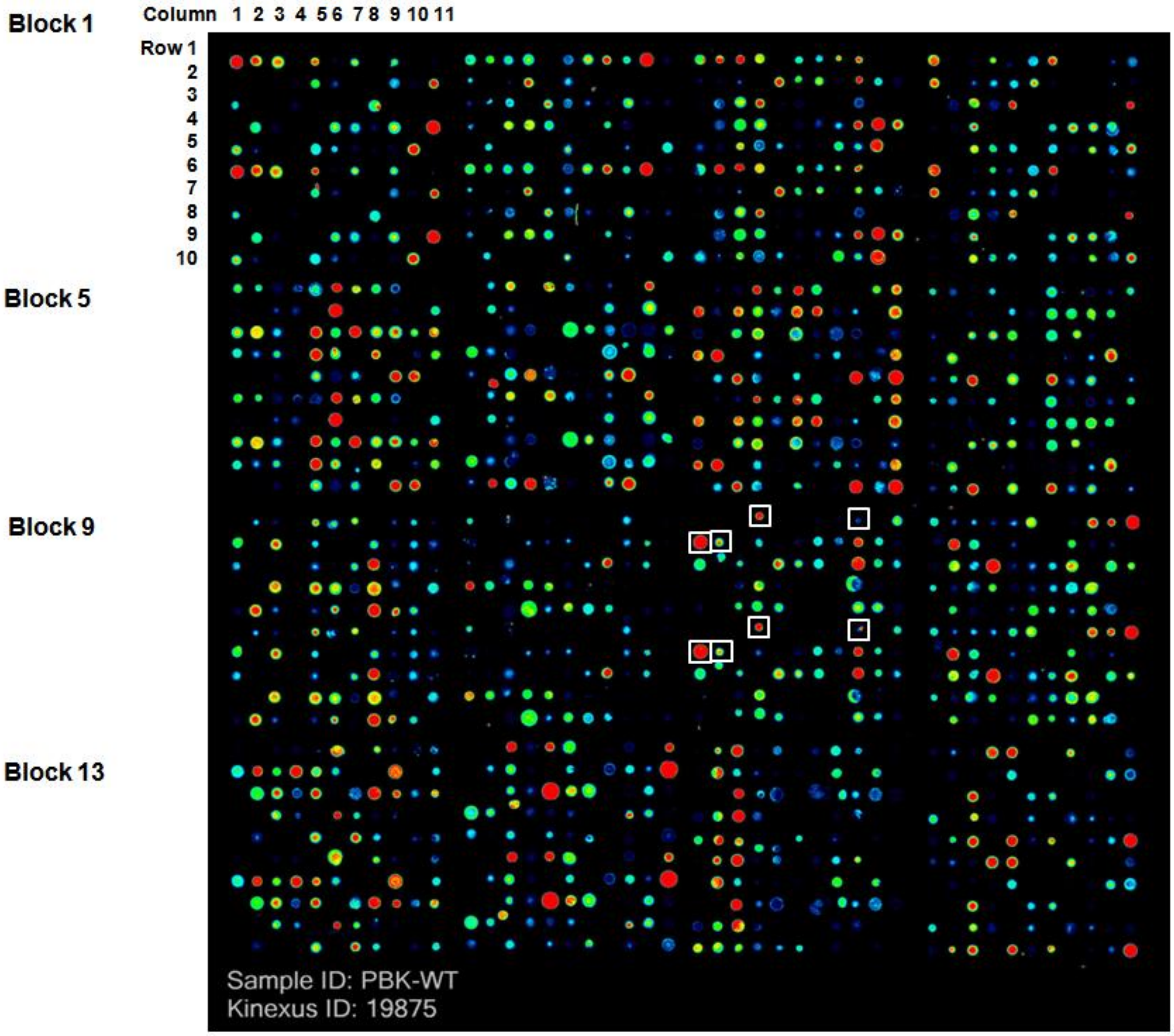

Figure S11. Full scanned-images of KAM-900P Antibody Microarray generated from the lysate of HEK293T cells expressing wild-type PBK. The spots of interest, taken to show in Figure 4, for p38a MAPK(T180+Y182), p38b MAPK(T180+Y182), p38d MAPK(Pan-specific), and p38d MAPK(Y182) are located at [Block, (Rows), Column]; [11, (1 and 6), 4], [11, (1 and 6), 9], [11, (2 and 7), 1], and [11, (2 and 7), 2] respectively, as show in squares.

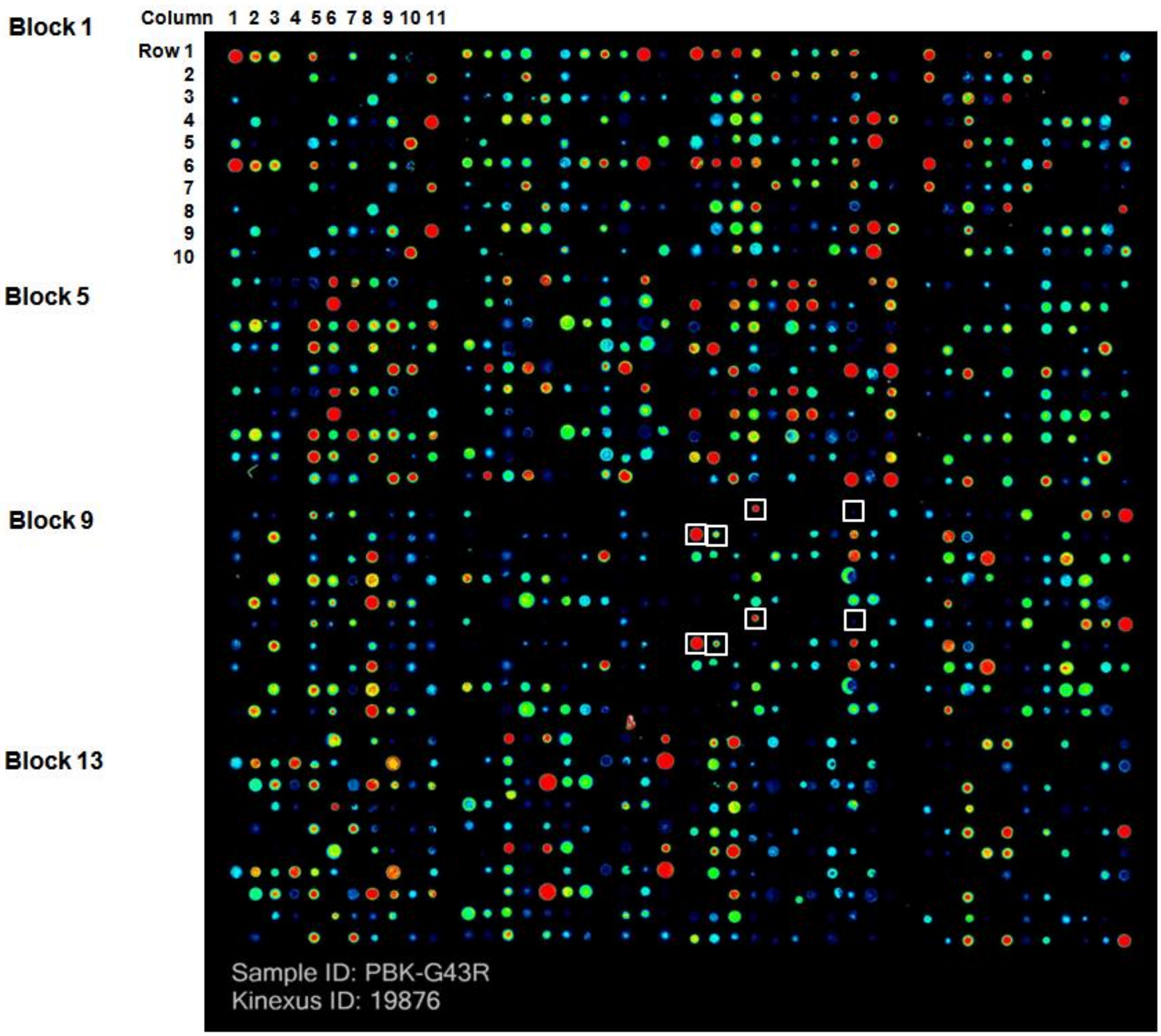

Figure S12. Full scanned-images of KAM-900P Antibody Microarray generated from the lysate of HEK293T cells expressing p.Gly43Arg PBK. The spots of interest, taken to show in Figure 4, for p38a MAPK(T180+Y182), p38b MAPK(T180+Y182), p38d MAPK(Pan-specific), and p38d MAPK(Y182) are located at [Block, (Rows), Column]; [11, (1 and 6), 4], [11, (1 and 6), 9], [11, (2 and 7), 1], and [11, (2 and 7), 2] respectively, as show in squares.

Supplementary tables

Table S1. Some characteristics of patients with kidney stone disease (KSD) and normal control subjects.

Characteristics	Patients (n)	%	Controls (n)	%
**Gender**				
Female	77	42.78	81	45.00
Male	103	57.22	99	55.00
**Age**				
Mean (years)	44.1 ± 12.8	-	44.3 ± 12.5	-
Range (years)	22-73	-	21-75	-
**Familial history**				
Yes	128	71.11	-	-
No	52	28.89	-	-
**Stone number**				
Single	50	27.78	-	-
Multiple	120	66.67	-	-
Unknown	10	5.55	-	-
**Position of stone**				
Kidney	163	90.55	-	-
Ureter	5	2.78	-	-
Kidney and ureter	12	6.67	-	-

Table S2. Blood and urine chemistry analyzed in proband (II:5) and his sister (II:1)

**Patient data**	**II:5**	**II:1**	**Normal range**
Gender	Male	Female	-
Age	48	59	-
Serum analysis			
Creatinine (Cr)	3.7	5.4	Male: 0.67-1.17, Female 0.51-0.95 mg/dL
Magnesium (Mg)	0.87	0.84	1.6-2.6 mg/dL
Calcium (Ca)	8.3	9.1	8.6-10.0 mg/dL
Sodium (Na)	139	140	136-145 mmol/L
Potassium (K)	4.38	4.11	3.4-4.5 mmol/L
Chlorine (Cl)	106	108	98-107 mmol/L
Phosphorus (P)	3.3	3.7	2.5-4.5 mg/dL
Urine analysis			
Creatinine (Cr)	108	-	Male: 40-278, Female 29-226 mg/dL
Magnesium (Mg)	1.4	-	4.10-13.80 mmol/L
Calcium (Ca)	7.2	-	6.8-21.3 mg/dL
Sodium (Na)	74	-	54-190 mmol/L
Potassium (K)	23	-	20-80 mmol/L
Chlorine (Cl)	75	-	46-168 mmol/L
Citrate (Cit)	1.77	-	
Phosphate (PO4)	65	-	40-136 mg/dL
Uric acid	18.5	-	2.2-275 mg/dL

Table S3. Prediction of the impact of amino acid changes on the protein structure and function of PBK resulting from the identified variations in exons of PBK using 6 web-based programs.

PCR-Fragment	Nucleotide changes (NM_018492.4)	Location	dbSNP	1000G Frequency	Effect	AA Change (NP_060962.2)	Prediction (6 programs)	Exonic splicing enhancer	Cryptic splice site
Ex1-2	c.-100G>A	5'UTR	rs3735743	0.024	-	-	-	Negative	Positive
	**c.-99C>A**	**5'UTR**	**rs3735744**	**0.024**	**-**	**-**	**-**	**Positive**	**Positive**
**Ex2**	**c.14G<C**	**Exon 2**	**-**	**-**	**Missense variant**	**p.Ser5Thr**	**0**	**Positive**	**Positive**
	c.58+16T>G	Intron 2	rs10113621	0.222	-	-	-	Negative	Positive
	c.58+91G>T	Intron 2	rs889216779	-	-	-	-	Negative	Negative
**Ex3**	**c.127G>A**	**Exon 3**	**-**	**-**	**Missense variant**	**p.Gly43Arg**	**5**	**Positive**	**Negative**
**Ex4**	**c.295+4C>A**	**Intron 4**	**rs727813**	**0.416**	**-**	**-**	**-**	**Positive**	**Positive**
Ex5	c.320A>G	Exon 5	rs3779620	0.222	Missense variant	p.Asn107Ser	0	Negative	Positive
Ex8-1	c.773-4C>G	Intron 7	-	-	-	-	-	Positive	Negative
	c.822G>C	Exon 8	rs2294092	0.419	-	p.Ala274Ala	-	Negative	Negative
Ex8-2	c.*103G>C	3'UTR	rs1052874	0.222	-	-	-	Positive	Negative
**Ex8-4**	c.*540_*541insCTT	3'UTR	-	-	-	-	-	Positive	Negative
	**c.*683_*685delAAG**	**3'UTR**	**-**	**-**	**-**	**-**	**-**	**Positive**	**Positive**

Table S4. Variants from the exome sequencing data of III:3 and III:4 in comparison to the other affected members over a set of 175 candidate genes reported to be involved in KSD.

No.	Gene Name	dbSNP	Nucleotide changes	Amino acid changes	Variant function
1	ADAMTS14	rs12774070	c.2818C>A	p.Leu940Met	Oral tumor cell differentiation and associated with liver tumorigenesis
2	BMS1	rs2272881	c.710G>A	p.Arg237His	No data
3	CASR	rs1801725	c.2986G>T	p.Ala996Ser	Associate with serum calcium level and KSD. Increases risk of more advanced sHPT and contributes to infection-related mortality in hemodialysis patients
4	CCDC157	rs740223	c.151G>A	p.Asp51Asn	No data
5	CCDC157	rs4820839	c.1504-6T>C	.	No data
6	CDH1	rs3743674	c.48+6C>T	.	No data
7	CLCN2	rs9820367	c.2003C>G	p.Thr668Ser	Associated with migrating partial seizures
8	CYP17A1	rs72559703	c.32C>T	p.Thr11Ile	No data
9	DGKH	rs139794584	n.2817+1G>A	.	No data
10	GLCCI1	-	c.473C>A	p.Ser158Tyr	No data
11	HSPG2	rs2270695	c.9332-7C>T	.	No data
12	HSPG2	rs2270696	c.9332-8C>T	.	No data
13	HSPG2	rs2290498	c.5705-5G>A	.	No data
14	IL1RN	rs878972	c.73+8A>C	.	No data
15	MAGI1	rs148924626	c.1546+5C>A	.	No data
16	MEFV	rs77380520	c.1759+8C>T	.	No data
17	MEFV	rs11466024	c.1223G>A	p.Arg408Gln	Associated with an incomplete FMF, PFMS and PFAPA syndrome.
18	MEFV	rs11466023	c.1105C>T	p.Pro369Ser	Associated with an incomplete FMF, PFMS and PFAPA Syndrome.
19	MEFV	rs224222	c.605G>A	p.Arg202Gln	Associated with FMF when it is in homozygous pattern or with another *MEFV* mutations
20	MEFV	-	c.238C>T	p.Arg80Cys	No data
21	MIF	rs2070766	c.282-6C>G	.	No data
22	MMP9	rs3918254	c.997+5C>T	.	Associated with HSPN and PACG
23	SLC12A3	rs11643718	c.2738G>A	p.Arg913Gln	Associated with albumin excretion and the diabetic nephropathy in Type 2 diabetes mellitus and Gitelman syndrome
24	SLC34A3	rs28407527	c.757T>C	p.Leu253Leu	No data
25	SLC4A1	rs5035	c.113A>C	p.Asp38Ala	An innocuous polymorphism and clinical significance remains unknown
26	ZNF365	-	c.260G>A	p.Ser87Asn	No data

sHPT = secondary hyperparathyroidism, FMF = familial mediterranean fever, PFMS = protracted febrile myalgia syndrome, HSPN = Henoch-Schönlein purpura nephritis, PACG = primary angle-closure glaucoma

Table S5. PCR primers for genotyping 3 genetic variations in *ANKRD36*, *PBK*, and *SORL1* genes in UBRS033 family members, 180 unrelated patients, and 180 normal control subjects.

Gene	Variation	Nucleotide sequence (5'→3')	Number of nucleotides	Tm (°C)	Product size (bp)
ANKRD36	p.Ala68Thr	AAAGTCCTGTCACTCTCACAGGAGC	25	64.5	
		GTACTTCAACCAAATCCATCTC	22	55.3	185
PBK	p.Gly43Arg	ATGCAGAAGCTTGGCTTTGG	20	62.7	
		AGCCAGGATCAGGAGCAGAC	20	61.9	172
SORL1	p.Arg1501Trp	GTGTATTCCCAACTGGAAGC	20	57.1	
		GAGAATGCAGTAAGAGCATGG	21	57.6	212

Table S6. PCR primers for the amplification of all exons of *PBK* for screening and sequencing of its genetics variations.

Fragments no.	Region	Primer name	Nucleotide sequence (5'→3')	Number of nucleotides	Tm (°C)	Product size (bp)
Ex1-1	Exon 1	F1F	CTCCTGGGGAGCAGATGTT	19	60.2	
		F1R	CCTCCCTCTCCCAGGACTC	19	61.2	392
Ex1-2	Exon 1	F2F	CTTCCTTCCCTCGTCTTTGG	20	61.1	
		F2R	AGATCCTCCCCTGTCACGAA	20	62.0	390
Ex2	Exon 2	F3F	GCCTGGCTATTGGCATTTTTC	21	62.9	
		F3R	GATCAAACCGTGGCCTATAAAA	22	60.2	335
Ex3	Exon 3	F4F	TGCTCTCCAAAAAGACTGTGC	21	60.6	
		F4R	AGGGTCTAGGGACCGGAATA	20	59.8	358
Ex4	Exon 4	F5F	TCCCAAAAATTTGGAAGTCA	20	58.0	
		F5R	AAATATCCAGCCCCATCCTC	20	60.1	339
Ex5	Exon 5	F6F	TCAGCGTTAGTCCAGATCCA	20	59.4	
		F6R	TCCATCCACATTCAAAGATG	20	56.8	369
Ex6	Exon 6	F7F	TGAAACGCTGGACAAATGTAA	21	59.2	
		F7R	TTTGATCCATGTGGACTTACG	21	58.5	339
Ex7	Exon 7	F8F	GTTCAAATCGTTCGCCTGTT	20	60.1	
		F8R	AACCTTGGTGGTACCAAATCA	21	59.2	383
Ex8-1	Exon 8	F9F	CTGTCTGTGGCAGAATGGAA	20	59.8	
		F9R	ACAATGTGTGCAGCAGAAGG	20	59.9	298
Ex8-2	Exon 8	F10F	TGCACTAATGAAGACCCTAAAGA	23	58.1	
		F10R	TGTTTTAAGTCAGCATGAGCAG	22	58.2	330
Ex8-3	Exon 8	F11F	TGGAATTGTACTGGGTTTTCTG	22	59.0	
		F11R	CCAAAGATCTGAGGAGATCCA	21	59.2	366
Ex8-4	Exon 8	F12F	CACTGAACCTTTTGCTGATGTG	22	60.7	
		F12R	GGCCAAGAGAGGGAAAGAAA	20	60.7	367

Table S7. PCR primers for analysis of *PBK* expression.

Gene	Nucleotide sequence (5'→3')	Number of nucleotides	Tm (°C)	Product size (bp)
PBK exon 5-6	TTTACTGAAGCCAATGATGG	20	56.3	
	TCCAGTGGTAGAGAGACTCC	20	54.2	275
PBK exon 7-8	GAAGCTGTGGAGGAGAATGGT	21	60.6	
	ACAATGTGTGCAGCAGAAGG	20	60.0	299
ACTB	GCTCGTCGTCGACAACGGCTC	21	62.3	
	CAAACATGATCTGGGTCATCTTCTC	25	55.6	353

Supplementary Methods

Exome sequencing

DNA samples of five affected members (II:1, II:3, II:4, III:3 and III:4) and three unaffected members (I:2, II:6 and II:7) from the UBRS033 family were sent to Macrogen (Seoul, South Korea) for exome sequencing analysis. Exome capture was performed using an Illumina TruSeq Exome Enrichment Kit (Illumina, San Diego, California, USA) or Sure Select V5+UTR, and sequencing was performed using an Illumina HiSeq 2000 or Illumina HiSeq 3500. The reads were mapped against UCSC hg19 (http://genome.ucsc.edu/) by Burrows-Wheeler Aligner (BWA, http://bio-bwa.sourceforge.net/), and variations (SNPs and Indels) were detected by Sequence Alignment/Map (SAMTOOLS, http://samtools.sourceforge.net/).

Genetic variations acquired from whole exome sequencing were further analyzed. The variations outside exonic regions were initially excluded because they are likely to be non-disease-causing polymorphisms. Since KSD in the UBRS033 family was inherited as autosomal dominant mode, the genetic variations that were shared in the five affected family members, but that were not observed in the unaffected family members, were selected for further analyses. The variations in exons that caused non-synonymous changes, stop gain/loss variants, or short insertions or deletions (Indels) were considered for further analysis.

Prediction of the impact of amino acid changes on protein structure and function

The impact of amino acid changes on protein structure and function was predicted using 6 web-based programs, including Polymorphism Phenotyping v2 (PolyPhen-2)[1], VarioWatch[2], MutationTaster[3], Sorting Intolerant From Tolerant (SIFT)[4], MutationAssessor[5], and Likelihood Ratio Test (LRT)[6]. The impact of exon-intron boundaries on the mRNA splicing process was evaluated using ESEfinder 2.0[7]. Multiple amino acid sequence alignment of the candidate genes from human, chimpanzee, orangutan,

gibbon, dog, cow, mouse, rat, anole, chicken and zebra fish were performed using Clustal Omega program (https://www.ebi.ac.uk/Tools/msa/clustalo/).

Genotyping of genetic variations in family members and normal control subjects

Nucleotide sequences of *ANKRD3*, *PBK*, and *SORL1* genes were acquired from the GenBank database for the purpose of designing polymerase chain reaction (PCR) primers (Supplementary Table S3). The specific primer-pairs generated amplicons ranging in size from 172 to 212 bp, covering the candidate variations for the genotyping of all available members in the UBRS033 family and all normal control subjects by PCR-high resolution melting (PCR-HRM) analysis[8,9] and either PCR-restriction fragment length polymorphism (PCR-RFLP) or derived cleaved amplified polymorphic sequences (dCAPS) method[10]. The genetic variations that were identified by these genotyping methods in all DNA samples were confirmed by Sanger DNA sequencing.

Segregation and haplotype analysis

The SNP genotyping data from PCR-RFLP or PCR-dCAP were analyzed by easyLINKAGE software[11]. We used SuperLink to calculate the linkage with parametric two-point method. Genome-Wide Human SNP Array 6.0 (Affymetrix, Santa Clara, CA, USA) was used to genotype 11 DNA samples of the UBRS033 family. The SNP genotyping data from both methods were analyzed by GeneHunter to determine the haplotypes and their segregation within the family.

Screening of genetic variations in PBK by PCR-HRM method

The nucleotide sequence of *PBK* was obtained from the GenBank database. Twelve pairs of PCR primers covering 8 exons and their exon-intron boundaries were designed (Supplementary Table S4) and synthesized. Genetic variations in *PBK* in 180 DNA samples from the patients with KSD were screened by PCR-HRM method using a LightCycler 480 II machine (Roche Diagnostics, Penzberg, Germany). The PCR mixture contained Resolight dye (Roche Diagnostics, Mannheim, Germany) for detection of PCR

products and for analysis of their melting curves. The melting curve was normalized for temperature-shifted difference plot by Gene Scanning 1.5.0 software (Roche Diagnostics, Penzberg, Germany). The samples with a melting profile different from the normal one were further analyzed by DNA sequencing.

DNA sequencing

To prepare PCR product for DNA sequencing, PCR reaction was performed in a volume of 50 μL containing 200 ng genomic DNA in 1x reaction buffer, 0.5 μM of each primer, 0.2 mM dNTP, 2.0 mM $MgCl_2$, and 1.25 units of DNA polymerase (HS Prime Taq DNA polymerase; GENET BIO, Chungcheongnam-do, South Korea) using a TProfessional standard thermocycler (Biometra, Goettingen, Germany). The PCR product was purified before processing for direct DNA sequencing by the Sanger method. The sequencing reaction was performed using BigDye™ Terminator by a service provider of First BASE Laboratories Sdn Bhd (Selangor, Malaysia). The sequencing data were analyzed via comparison with a reference nucleotide sequence by multiple sequence alignment using Clustal Omega program (https://www.ebi.ac.uk/Tools/msa/clustalo/).

Plasmid constructs, cell culture, and transfections

Wild-type (WT) *PBK* was generated using human kidney cDNA as a template for amplification of *PBK* coding sequences. The PCR fragment contained *Kpn*I and *Xho*I recognition sites for insertion into pcDNA™ 3.1 (+) vector. The wild-type construct was used as a template to generate mutant (G43R) construct by PCR and site-directed mutagenesis method using *Pfx* DNA polymerase (Invitrogen, Carlsbad, CA, USA). All constructs were confirmed by Sanger DNA sequencing (First BASE Laboratories Sdn Bhd, Selangor, Malaysia).

HEK293T cells were cultured in complete Dulbecco's Modified Eagles Medium (DMEM; Gibco Life Technologies, Waltham, MA, USA) supplemented with 10% (v/v) fetal bovine serum, and 1.2% (v/v) penicillin-streptomycin solution (100 U/ml penicillin and 100 μg/ml streptomycin) in a humidified CO_2 (5%) incubator at 37°C. One day before transfection, the HEK293T cells were collected by

trypsinization and seeded in 6-well plates. The HEK293T cells were transfected with pcDNA™ 3.1 or WT or G43R plasmid constructs using Lipofectamine™ 2000 (Invitrogen) according to the manufacturer's instructions. After transfection, the cells were incubated for an additional 24 hours prior to further investigations.

Reverse transcription and polymerase chain reaction (RT-PCR)

Total RNA was extracted from human fresh frozen kidney tissues, HEK293 cells, and HEK293T cells using Trizol reagent (Invitrogen) according to the manufacturer's protocol. RNA was then reverse-transcribed into cDNA using Oligo d(T) and SuperScript™ III First-Strand Synthesis System (Invitrogen, California, USA). *PBK* cDNA sequence was analyzed by amplifications of two regions, including exons 5-6 and exons 7-8, and *ACTB* cDNA was amplified to serve as an internal control. The sequences of primers are shown in Supplementary Table S5.

Western blot analysis

Total proteins were extracted from human kidney tissue (20 µg) or cell line ($1x10^8$cell) with RIPA buffer. Twenty µl of human kidney tissue or cell line protein was separated by SDS-polyacrylamide gel electrophoresis (PAGE). After electrophoresis, the proteins were transferred onto a nitrocellulose membrane following a standard protocol. After blocking with 5% skim milk powder, the membrane was incubated with mouse anti-human PBK (sc-293028; Santa Cruz Biotechnology, Dallas, TX, USA) at a dilution of 1:500, followed by incubation with rabbit anti-mouse antibody conjugated-HRP (Dako Cytomation, Glostrup, Denmark) at a dilution of 1:1,000. For detection of transfected PBK protein, the membrane was incubated with mouse anti-FLAG M2 antibody (Sigma-Aldrich, St Louis, MO, USA) at a dilution of 1:3,000, followed by incubation with rabbit anti-mouse antibody conjugated-HRP (Dako Cytomation) at a dilution of 1:1,000. For downstream signaling analysis, rabbit anti-total p38 MAPK (sc-535; Santa Cruz Biotechnology) at a dilution of 1:1,000 or mouse anti-phosphorylated p38 MAPK (sc-7973; Santa Cruz Biotechnology) at a dilution of 1:500 was used, followed by incubation with rabbit anti-

mouse antibody conjugated-HRP or swine anti-rabbit antibody conjugated-HRP (Dako Cytomation) at a dilution of 1:1,000.

Human GAPDH in each sample was detected as an internal control using mouse anti-GAPDH antibody (Santa Cruz Biotechnology) at a dilution of 1:5,000, and rabbit anti-mouse antibody conjugated-HRP (Dako Cytomation) at a dilution of 1:1,000. Chemiluminescent signals generated by SuperSignal West Pico Chemiluminescent Substrate (Thermo Fisher Scientific, Waltham, MA, USA) were detected using a G:BOX Chemiluminescence Imaging System (Syngene, Cambridge, UK).

Human kidney immunohistochemistry (IHC) and immunofluorescence (IFA)

Fresh human kidney tissues were fixed with 4% formalin buffer and then paraffin-embedded to obtain 4-μm thick cross-sections of the kidney. After deparaffinization and rehydration in ethanol series, kidney sections were treated in sodium citrate buffer (10 mM sodium citrate, 2 mM EDTA, 0.05% Tween-20, pH 6.0) for retrieval of antigenic sites followed by blocking of endogenous HRP using 3% hydrogen peroxide. After blocking in 1% BSA, primary antibodies, including mouse anti-PBK at 1:25 (sc-293028; Santa Cruz Biotechnologies), rabbit anti-AQP1 at 1:200, rabbit anti-AQP2 at 1:50, mouse anti-V-ATPase at 1:25 (sc-55544; Santa Cruz Biotechnologies), and rabbit anti-Na^+/K^+-ATPase at 1:50 (EP1845Y; Abcam), were applied overnight at 4ºC. Secondary antibody conjugated-HRP at 1:250 (Dako, Japan) was applied for 2 h. Kidney sections were developed in 3% 3,3'-diaminobenzidine (DAB) followed by counterstaining with hematoxylin. Slides were captured by Axio Star Plus Light Microscope (Carl Zeiss Microscopy, Jena, Germany). For IFA, goat anti-mouse alexa-488 (Invitrogen) and donkey anti-Rabbit alexa-555 secondary antibodies at 1:200 (Invitrogen) were applied with DAPI staining (Molecular Probes, Eugene, OR, USA). Kidney sections were examined by using an LSM 800 Confocal Microscope (Carl Zeiss). Rabbit and mouse IgG1 (Cell Signaling Technology, Danvers, MA, USA) were used as isotype control in concentrations equal to those of the primary antibodies being used.

Stability of wild-type and p.Gly43Arg proteins expressed in HEK293T cells

The wild-type and p.Gly43Arg PBK proteins were overexpressed by transient transfection in HEK293T cells. After 24 hours of transfection, 100 μg/ml of cycloheximide (CHX) were treated for 0, 6, 12, 24, and 36 hours. The transfected HEK293T cells were lysed with RIPA buffer. Samples were centrifuged at 15,000 g for 20 min at 4°C, and the supernatant fraction was collected as total protein extraction. The wild-type and p.Gly43Arg PBK proteins were detected by immunoblotting method and were quantified by ImageJ 1.50i[12] and plotted as relative intensities.

Supplementary references

1 Adzhubei, I. A. et al. A method and server for predicting damaging missense mutations. Nature methods 7, 248-249, doi:10.1038/nmeth0410-248 (2010).

2 Cheng, Y. C. et al. VarioWatch: providing large-scale and comprehensive annotations on human genomic variants in the next generation sequencing era. Nucleic acids research 40, W76-81, doi:10.1093/nar/gks397 (2012).

3 Schwarz, J. M., Rodelsperger, C., Schuelke, M. & Seelow, D. MutationTaster evaluates disease-causing potential of sequence alterations. Nature methods 7, 575-576, doi:10.1038/nmeth0810-575 (2010).

4 Kumar, P., Henikoff, S. & Ng, P. C. Predicting the effects of coding non-synonymous variants on protein function using the SIFT algorithm. Nature protocols 4, 1073-1081, doi:10.1038/nprot.2009.86 (2009).

5 Reva, B., Antipin, Y. & Sander, C. Predicting the functional impact of protein mutations: application to cancer genomics. Nucleic acids research 39, e118, doi:10.1093/nar/gkr407 (2011).

6 Chun, S. & Fay, J. C. Identification of deleterious mutations within three human genomes. Genome research 19, 1553-1561, doi:10.1101/gr.092619.109 (2009).

7 Cartegni, L., Wang, J., Zhu, Z., Zhang, M. Q. & Krainer, A. R. ESEfinder: A web resource to identify exonic splicing enhancers. Nucleic acids research 31, 3568-3571 (2003).

8 Nettuwakul, C., Sawasdee, N. & Yenchitsomanus, P. T. Rapid detection of solute carrier family 4, member 1 (SLC4A1) mutations and polymorphisms by high-resolution melting analysis. Clinical biochemistry 43, 497-504, doi:10.1016/j.clinbiochem.2009.12.010 (2010).

9 Reed, G. H., Kent, J. O. & Wittwer, C. T. High-resolution DNA melting analysis for simple and efficient molecular diagnostics. Pharmacogenomics 8, 597-608, doi:10.2217/14622416.8.6.597 (2007).

10 Neff, M. M., Neff, J. D., Chory, J. & Pepper, A. E. dCAPS, a simple technique for the genetic analysis of single nucleotide polymorphisms: experimental applications in Arabidopsis thaliana genetics. The Plant journal : for cell and molecular biology 14, 387-392 (1998).

11 Lindner, T. H. & Hoffmann, K. easyLINKAGE: a PERL script for easy and automated two-/multi-point linkage analyses. Bioinformatics 21, 405-407, doi:10.1093/bioinformatics/bti009 (2005).

12 Schneider, C. A., Rasband, W. S. & Eliceiri, K. W. NIH Image to ImageJ: 25 years of image analysis. Nature methods 9, 671-675 (2012).
